# A highly fluorescent bora[6]helicene exhibiting circularly polarized light emission†

**DOI:** 10.1039/d3sc05171d

**Published:** 2024-01-05

**Authors:** Matthias Schnitzlein, Kazutaka Shoyama, Frank Würthner

**Affiliations:** a Universität Würzburg, Institut für Organische Chemie, Center for Nanosystems Chemistry Am Hubland 97074 Würzburg Germany wuerthner@uni-wuerzburg.de

## Abstract

Heteroatom-doped helicenes have attracted great research interest due to their inherent chirality enabling fascinating new applications. Herein we present our successful synthesis of 19c-boratribenzo[*gh*,*jk*,*mn*][6]helicene, the hitherto longest and first configurationally stable pristine bora[*n*]helicene. It displays intense orange fluorescence and circularly polarized light (CPL) emission with a high quantum yield of up to 84%. X-ray single crystal analysis reveals a highly twisted, helical shape and intriguing intermolecular stacking. Complexation with a size-complemental aza[4]helicene yielded an unprecedented hetero-chiral π–π-stacked helicene dimer.

## Abstract 2

Synthesis and characterization of the first configurationally stable pristine boron-doped helicene, a bora[6]helicene, is described.

## Graphical abstract

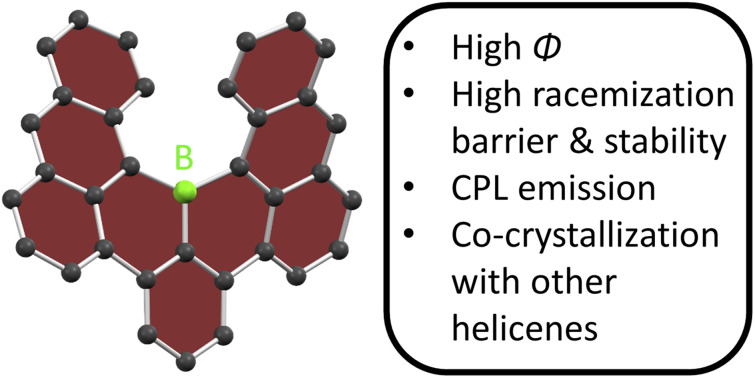

## Introduction

**Fig. 1:**
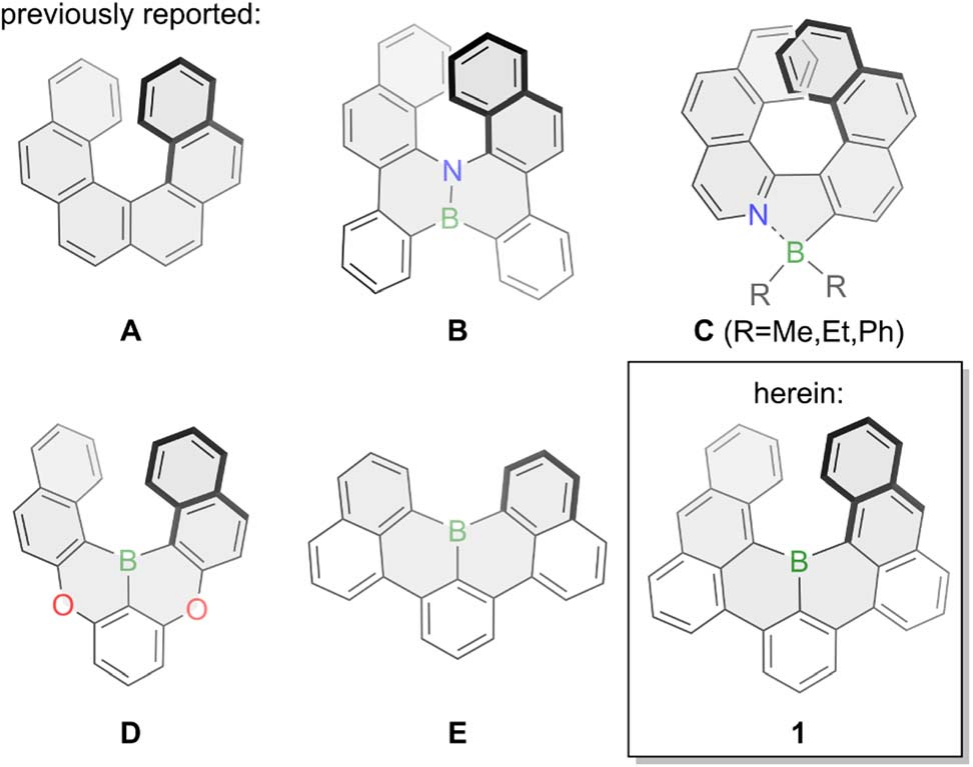
Previously reported boron-doped helicenes and bora[6]helicene 1 presented herein.

Phenanthro[3,4-*c*]phenanthrene (A, Fig. 1) – more commonly named [6]helicene – is a non-planar polycyclic aromatic hydrocarbon (PAH) that is bent into a helical shape due to overcrowding of its six *ortho*-fused benzene rings. Its defining structural element is a corkscrew-like structure with a *C*_2_-axis that renders it chiral even though it does not have any asymmetric carbons. The first synthesis of [6]helicene was reported in 1956 by Newman and coworkers,^1,2^ almost three decades after the synthesis of its smaller congener [5]helicene was reported by Weitzenböck.^3^ Since then a large number of helicenes and helicenoids have been synthesized and investigated, and particularly in the last decade scientific interest skyrocketed.^4–8^ Their inherent chirality enables chemists to study various phenomena such as circular dichroism (CD) and circularly polarized light (CPL) emission with especially the latter enabling fascinating new applications. CPL materials are characterized by their fluorescence quantum yield *Φ* referring to their ratio of emitted *versus* absorbed photons, and their luminescence dissymmetry factor *g*_lum_ indicating the intensities of left- or right-handed polarized light with values between −2 and +2.^8^ Pristine carbohelicenes such as [6]helicene A typically have very low fluorescence quantum yields (*Φ*(A) = 0.04 in dioxane) with a downwards trend upon elongation of the scaffold^9^ and *g*_lum_ values in the 10^−4^ to 10^−2^ range.^10^

To overcome those limitations chemists usually resolve to heteroatom doping by inserting main-group elements such as boron, silicon, oxygen, nitrogen or phosphorous into the helical scaffold or by grafting those heteroatoms onto it. Boron-doping of helical PAHs especially allows for efficient tailoring of their optical and electronic properties due to boron's vacant p_*z*_ orbital as well as the introduction of a permanent dipole moment.^11,12^ Due to the inherent reactivity of three-coordinate organoboron compounds towards oxygen and moisture the synthesis of pristine boron-doped helicenes is underdeveloped and most commonly stabilizing nitrogen or oxygen atoms are introduced into the helicene scaffold, aptly named aza- or oxaborahelicenes. This is exemplified by the synthesis of semi-conducting azabora[6]helicene B in 2012.^13^ Similarly, nitrogen-coordination onto four-coordinate boron was also a successfully employed strategy as shown by the group of Nowak-Król for azaborahelicene C in 2021.^14^ Further examples for azaborahelicenes were reported by the groups of Staubitz^15^ and Ingleson.^16^ The Hatakeyama group also succeeded in the synthesis of oxabora[6]helicene D in 2015 with a 1,4-oxabora substitution pattern enabling thermally activated delayed fluorescence (TADF).^17^ Additionally, doping of azaborahelicenes with additional sulfur was also reported by the group of Yang.^18^ Conversely, pristine borahelicenes are much rarer with to the best of our knowledge only small bora[4]helicenes reported such as E which was first synthesized in 2015 by two different approaches from the groups of Hatakeyama and Wagner.^19,20^ It displayed high stability whilst delivering bright green fluorescence with a very high fluorescence quantum yield of 90% in toluene.^20^ A similar compound with a bora[4]helicene unit was more recently synthesized by the group of Feng.^21^ Whereas its optical properties are excellent, its small [4]helicene moiety results in a very low racemization barrier, thereby negating its use as chiral emitter.

Intrigued by the facile synthesis strategy employed by the group of Hatakeyama, the promising stability and the optical properties of E,^19,20^ we envisioned the synthesis of bora[6]helicene 1 to shed light onto pristine boron-doped chiral emitting PAHs. Theoretical calculations at the B3LYP/6-31+G(d) level of theory attributed a racemization barrier of around 35 kcal mol^−1^ to 1 (Fig. S24†) in line with pristine [6]helicene A (35.6 kcal mol^−1^),^22^ and sufficiently high for application as CPL emitter. Herein, we present the synthesis and properties of the hitherto longest boron-doped [*n*]helicene, bora[6]helicene 1. A *C*_2_-symmetry boron-doping of the [6]helicene scaffold endowed it with chiral emitting properties and up to 84% fluorescence quantum yield in toluene solution, which is a 21-fold increase compared to its pure-carbon analogue [6]helicene (*Φ* = 0.04 in dioxane).^9^ Further, we could successfully template the stable helical scaffold of 1 with a size-complementary aza[4]helicene^23^ yielding an unprecedented hetero-chiral helicene dimer.

## Results and discussion

### Synthesis

**Scheme 1:**
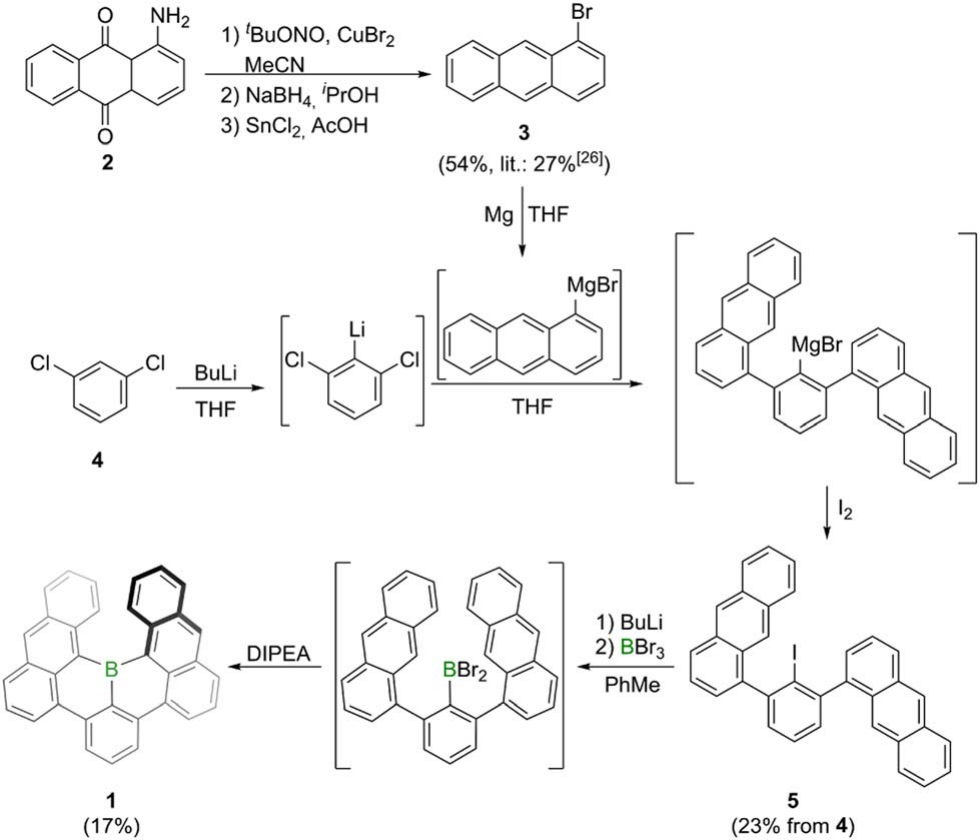
Synthesis of bora[6]helicene 1 by one-pot lithiation, borylation and intramolecular bora-Friedel–Crafts reaction. Precursor 3 synthesized by adapted literature methods.^26^ DIPEA = *N*,*N*′-diisopropylethylamine, THF = tetrahydrofuran.

Synthesis of borahelicene 1 was accomplished in two-steps from commercially available *m*-dichlorobenzene 4 (Scheme 1). The first step involved an adapted procedure used by Saednya and Hart for the synthesis of *meta*-terphenyls.^24^ Thus, 4 was selectively lithiated at −78 °C in THF by treatment with *n*-butyllithium and subsequently reacted with freshly prepared 1-anthracenyl magnesium bromide. After stirring for 36 h at 80 °C the magnesium bromide intermediate was quenched by addition of iodine to furnish 5 in 23% yield after column chromatography. According to previous work of Hart and coworkers this reaction most likely proceeds *via* an elimination–addition aryne mechanism.^25^ The bisanthracenyl iodide 5 is rotationally constricted leading to both anthracene moieties not being chemically equivalent in the NMR spectrum. This steric demand may also be the reason for the rather modest yields as it hinders both the nucleophilic attack onto the *in situ* generated aryne species as well as the rotation of the anthracene moiety in the final borylation step. The borylation involved a similar approach as that utilized by the Hatakeyama group for the synthesis of E.^20^ Thus, 1 was furnished by lithium-iodide exchange with *n*-butyllithium in toluene, followed by BBr_3_-mediated borylation and subsequent intramolecular arene borylation with Hünig's base (DIPEA) as hydrogen bromide scavenger. After column chromatography and washing with *n*-hexane 1 was obtained as a purple solid in 17% yield. The solid is stable under ambient conditions for temperatures up to around 480 °C (Fig. S16†). Its solutions are also stable under ambient conditions when kept in the dark, otherwise slow decomposition (half-life time of around one week) was noted in micromolar chloroform solutions (Fig. S17†). Solutions of 1 in *ortho*-dichlorobenzene exhibit a half-life time of around one day when heated to 150 °C under air and daylight, whereas they are significantly more stable under nitrogen atmosphere and daylight (half-time life of around four days, Fig. S18†). The high stability of 1 could be attributed to delocalization of LUMO orbital throughout the whole molecular scaffold including the boron center, which makes the empty p_*z*_ orbital of the boron center less reactive (Fig. S20†).

### (Chir)optical properties

**Fig. 2:**
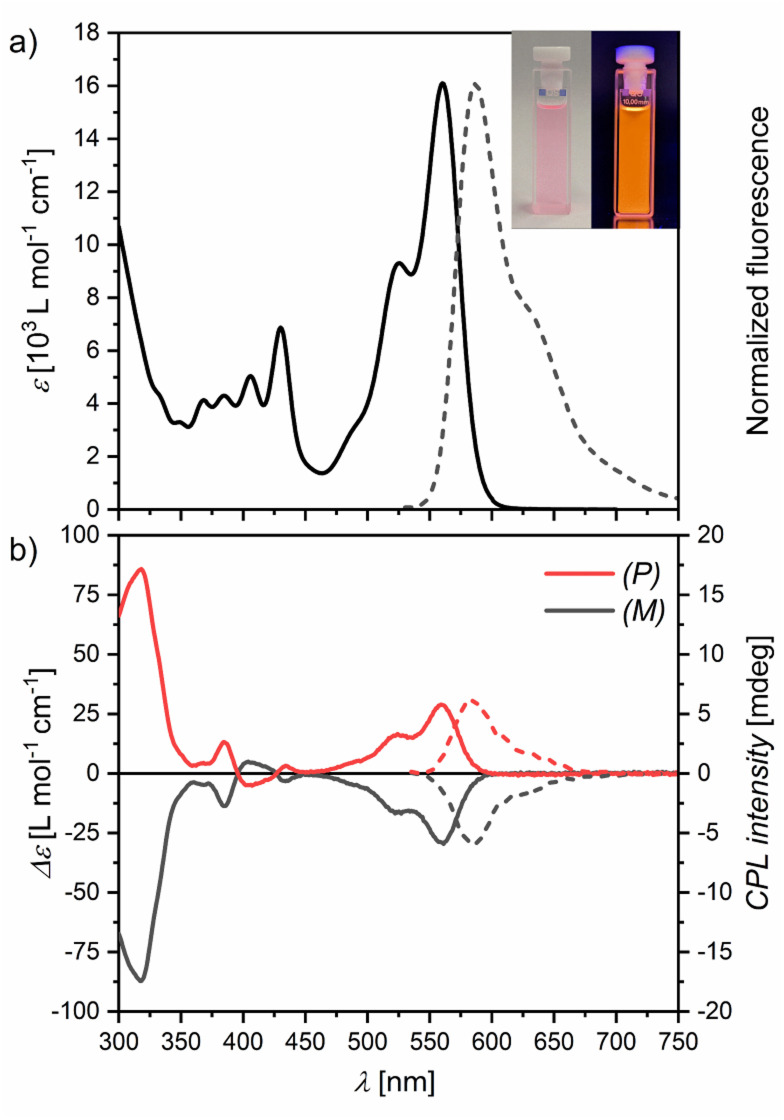
(a) UV/Vis absorbance (solid line) and fluorescence (dashed line) spectra of 1 in chloroform at 298 K. Inset are pictures of a chloroform solution of 1 under ambient (left) and UV (365 nm, right) light. (b) CD (solid lines) and CPL (dashed lines) spectra.

Bora[6]helicene 1 appears pink in solutions with intense orange fluorescence (Fig. 2a inset). UV/Vis absorption and fluorescence spectroscopy in chloroform (Fig. 2a) revealed moderate absorption in the visible wavelength regime with a maximum at 560 nm and a vibronic shoulder at 525 nm with absorption coefficients in the low 10^4^ M^−1^ cm^−1^ range. This absorption band could be assigned to the S_0_–S_1_ transition by TD-DFT calculations (Fig. S21†). Upon excitation mirror-like orange fluorescence with a small Stokes' shift of only 800 cm^−1^ and a maximum at 587 nm with a full width at half-maximum (FWHM) of 56 nm (1600 cm^−1^) was observed. A high fluorescence quantum yield (*Φ*) of 77% was measured in degassed chloroform with long life times (*τ*) of 10.1 ns (in aerated chloroform *Φ* = 70% with *τ* = 9.5 ns). Switching to degassed toluene as solvent afforded an increase in quantum yield to 84% (in aerated toluene *Φ* = 82%), which is comparable to that reported for E in the same solvent (90%).^20^ For degassed tetrachloromethane solutions a quantum yield of 81% was measured (in aerated tetrachloromethane *Φ* = 80%). Other emission properties were very similar for all three solvent systems with only minimal changes (Table S1 and Fig. S8 and S9†).

To study its chiroptical properties racemic 1 was separated by chiral HPLC (Fig. S14†). The first fraction could be assigned to the (*P*) enantiomer by its CD spectrum and TD-DFT calculations (Fig. S22†). Experimental racemization studies at 423 K in *ortho*-dichlorobenzene (*o*-DCB) confirmed the previously calculated racemization barrier of around 35 kcal mol^−1^ (Fig. S15†). This value is in line with carbo[6]helicene A (35.6 kcal mol^−1^)^22^ and azaborahelicene C (34.1 kcal mol^−1^),^14^ significantly higher than that of oxaborahelicene E (26.6 kcal mol^−1^)^27^ and slightly lower than the barrier of azaborahelicene B (calculated barrier 42.0 kcal mol^−1^).^13^ Similar to carbohelicenes, the racemization of 1 is hampered by the overcrowding of the outer benzenes of the two anthracenes, which leads to a transition state with the *C*_s_ symmetry with very close distances between adjacent hydrogens and thus to large strain energies (Fig. S24†). Both enantiomers show mirrored CD spectra (Fig. 2b) with Cotton effects especially strong in the UV region (*g*_abs_ = 1.5 × 10^−2^) and at their S_0_–S_1_ transition (*g*_abs_ = 1.9 × 10^−3^). Further, both enantiomers show mirrored CPL emission upon excitation at 430 nm (Fig. 2b). In line with the findings of Tanaka *et al.* for a wide range of organic chiral emitters,^28^ the *g*_lum_ values of 1 are slightly smaller than their corresponding *g*_abs_ values with a value of 1.4 × 10^−3^ as is typical of small organic molecules.^28,29^

### Crystallography

**Fig. 3:**
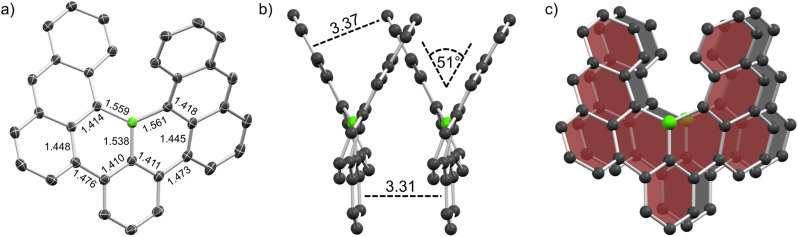
Solid state structure of racemic 1. (a) Crystal structure with ellipsoids set at 50% probability. (b and c) Homochiral slipped π-stack. Select bond lengths and distances are given in Å. Hydrogen atoms are omitted for clarity.

Crystals of racemic 1 and enantiopure (*M*)-1 were grown by physical vapor transport (PVT) and slow diffusion of acetonitrile into chlorobenzene solution, respectively, and were then studied by synchrotron X-ray diffraction. The crystal structure of 1 (Fig. 3a) closely matches calculations and as expected, the central boron adopts a trigonal planar geometry with angles of 114.76, 114.36 and 130.88°, respectively (sum of all angles 360.0°), and the steric overcrowding leads to its helical shape. The helix exhibits a tilt of 51° which is markedly lower than that of carbo[6]helicene (59°). The scaffold features B–C distances between 1.538 and 1.561 Å which are typical for single bonds between trigonal boron and sp^2^ carbon (B–C_ar_ = 1.556 Å).^30^ Similarly, the bond lengths between the central benzene moiety and both outer anthracene units were measured as 1.473 and 1.476 Å which is also in line with values obtained for biphenyl single bonds (C_ar_–C_ar_ = 1.487 Å).^30^ Accordingly, nucleus-independent chemical shift (NICS(1)_*zz*_)^31^ and anisotropy of the induced current density (ACID)^32^ calculations of 1 demonstrate semi-global aromaticity in the anthracene moieties and localized one in the central benzene ring interconnected by non-aromatic BC_5_ rings (Table S5 and Fig. S19†).

Contrary to B, which exhibited vastly different crystal packing in the racemate and its (*P*)-enantiomer that lead to p- to n-type charge carrier inversion,^13^ for 1 and (*M*)-1 no such differences were observed. They only differ in their space group: whereas the racemate features centrosymmetric *P*2_1_/*c*, the enantiopure crystal (chirality was assigned by CD spectra and TD-DFT calculations) exhibits non-centrosymmetric *R*3. However, both crystals feature homochiral slipped stacks (Fig. 3b and c) with close intermolecular distances of 3.31 and 3.37 Å between the central benzene and the outer anthracene moieties, respectively. These distances in conjunction with the offset stacking strongly hint at crystallization being driven by intermolecular π–π interactions.^33,34^ Such an arrangement presents a great opportunity for tuning of anisotropic properties as recently shown by Wade *et al.* for a dicyano[6]helicene whose orientation could be controlled by (in)organic templating into helical columns resulting in different responses towards circularly polarized light.^35^

**Fig. 4:**
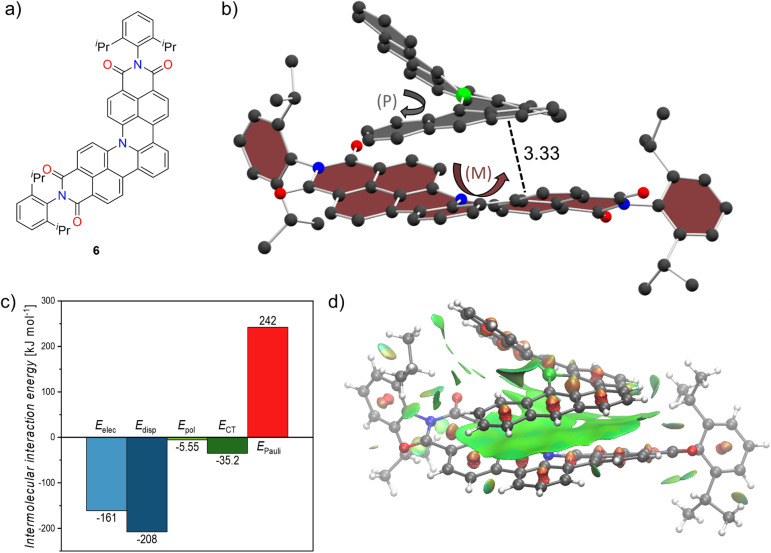
(a) Structure of templated dicarboximide embedded aza[4]helicene 6 used for cocrystallization. (b) Heterochiral cocrystal of 1 and 6. Hydrogens omitted for clarity, distances given in Å. (c) Graph of ALMO-EDA (B3LYP-D3/6-311+G(d,p)) with individual electrostatic (*E*_elec_), dispersion (*E*_disp_), polarization (*E*_pol_), charge-transfer (*E*_CT_) and Pauli-repulsion (*E*_Pauli_) energies. (d) Plot of non-covalent interaction surface. Green: dispersion interaction surface, red: steric clashes.

Inspired by the non-planar boron-doped scaffold of 1 and its non-covalent interactions we attempted co-crystallization with a non-planar nitrogen-doped molecule 6 which we recently reported (Fig. 4a)^23^ in order to induce chirality in an otherwise rapidly racemizing [4]helical scaffold. By slow diffusion of *n*-hexane into a stoichiometric mixture of racemic 1 and 6 in toluene we were able to obtain co-crystals which were subsequently studied by synchrotron X-ray diffraction. The co-crystals exhibit the *P*2_1_ space group and formed size-complemental hetero-chiral stacks as shown in Fig. 4b for an exemplary (*P*)-1/(*M*)-6 pair. A moderate distance of 4.04 Å was found between the nitrogen atom of the aza[4]helicene and the boron atom of bora[6]helicene. Along with the scaffold retaining the trigonal planar arrangement of the boron center these observations hint at weak B–N interactions. This is also supported computationally by plotting the non-covalent interaction (NCI) surface (*vide infra*). There is extensive and offset stacking between the outer π-surfaces present with interplanar distances of 3.33 Å. Due to their heterochirality only one half of each molecules' helix exhibits attractive dispersion interactions whereas the other is simply pointed away to not collide with the other helix. For further insight into the non-covalent interactions, we performed absolutely localized molecular orbital energy decomposition analyses (ALMO-EDA). Thereby, the total intermolecular interaction *E*_tot_ is decomposed into the individual electrostatic (*E*_elec_), dispersion (*E*_disp_), polarization (*E*_pol_), charge-transfer (*E*_CT_) and Pauli-repulsion (*E*_Pauli_) components.^36^ Dispersion interactions predominate in the stabilizing forces followed closely by electrostatics, supporting the close interplanar distances between the two π-systems. Polarization and charge transfer on the other hand, have an almost negligible contribution to stabilizing the cocrystal (Fig. 4c). We further probed these interactions by calculating the non-covalent interaction (NCI) surface.^37^ As plotted with vmd^38^ in Fig. 4d, the surface spans the whole of the bora[6]helicene scaffold indicating favorable interactions across the whole π-surface including the vicinity around the boron atom of 1 and the nitrogen atom of 6. We thus proved that our bora[6]helicene is able to successfully induce chirality in a [4]helicene derivative and envision to use this strategy to furnish tailor-made anisotropic chiral materials.

### Electrochemical measurements

To probe the electronic properties of 1 we conducted differential pulse and cyclic voltammetry in dichloromethane with 0.1 M *n*-Bu_4_NPF_6_ as electrolyte and the Fc/Fc^+^ couple as reference (Fig. S6†). As expected, the boron-doping leads to a rather facile reduction at −1.58 V that is anodically shifted compared to the smaller [4]borahelicene E (−1.91 V in *o*-DCB,^19^ −1.76 V in acetonitrile^20^). Conversely, the reduction of 1 is irreversible. Interestingly, 1 also features very low-lying oxidation potentials at +0.66 and +0.96 V. We attribute these to oxidation of the anthracene unit at its center. In a similar fashion, the group of Yamaguchi could already use adjacent boron to stabilize carboradicals.^39,40^ These redox values add up to an electronical gap of 2.24 eV. This is in line with the optical S_0_–S_1_ transition at 560 nm (2.21 eV) and the DFT-calculated HOMO–LUMO gap (2.51 eV). Calculations at the B3LYP/6-311+G(d,p) level of theory attest a HOMO energy of −5.19 eV and show that it is overwhelmingly localized on both anthracene moieties. For the LUMO however, an energy of −2.69 eV is obtained with localization expectedly centered on boron and smaller contributions of both anthracene units (Fig. S20†).

## Conclusion

To summarize, herein we present the successful synthesis of the hitherto longest boron-doped [*n*]helicene, bora[6]helicene 1. This boron-doped PAH features outstanding emitting properties with intense orange fluorescence and 84% quantum yield in toluene. Its racemization barrier is sufficiently high for its use as chiral emitter and it shows CPL emission with a *g*_lum_ of around 1 × 10^−3^. Investigations of enantiopure and racemic single crystals by X-ray diffraction revealed close intermolecular interactions in a slip-stacked fashion. Furthermore this chiral bora[6]helicene could be complexed with a shape-complementary nitrogen-doped helicene, which resulted in a hetero-chiral complex. These intriguing features and its inherent chirality may enable fascinating applications and further studies are currently underway in our laboratory.

## Data availability

The experimental procedures, analytical data and computational details are available within the manuscript and its ESI.† CCDC numbers 2279526–2279528 contain the supplementary crystallographic data for 1, (*M*)-1 and the 1·6 co-crystal, respectively. Additional data underlying this study are openly available in Zenodo, see https://doi.org/10.5281/zenodo.8120581.

## Author contributions

M. S.: conceptualization, investigation, formal analysis, visualization, writing – original draft; K. S.: conceptualization, investigation (crystallography), formal analysis (crystallography), supervision, writing – review & editing; F. W.: conceptualization, supervision, writing – review & editing, funding acquisition.

## Conflicts of interest

There are no conflicts to declare.

## Supplementary Material

SC-015-D3SC05171D-s001

SC-015-D3SC05171D-s002
