# Supplementary material for: A highly fluorescent bora[6]helicene exhibiting circularly polarized light emission
Source: Chem Sci. 2024 Jan 5;15(8):2984–9. doi: 10.1039/d3sc05171d (PMC10882488; doi:10.1039/d3sc05171d)
Supplement: SC-015-D3SC05171D-s001 [file SC-015-D3SC05171D-s001.pdf]

Electronic Supplementary Material (ESI) for Chemical Science.
This journal is © The Royal Society of Chemistry 2024

Table of Contents

1. Materials and methods 2
2. Experimental procedures 3
3. NMR spectra 5
4. Cyclic voltammetry 7
5. UV/Vis absorption and fluorescence spectra 8
6. CD/CPL spectra 11
7. MALDI-TOF-MS spectra 12
8. Chiral separation and racemization studies 13
9. Stability measurements 14
10. Crystal structure data 15
11. Computational data 17
11.1. NICS and ACID data 17
11.2. Frontier molecular orbitals 18
11.3. TD-DFT data 19
11.4. Racemization barrier 20
11.5. Optimized structures 21
12. References 22

1. Materials and methods

General remarks: Commercially available chemicals were purchased and directly used without further purification. Preparative column chromatography was performed with glass columns of various sizes charged with silica-gel from Macherey-Nagel (particle size 40–63 µm) as stationary phase. Dichloromethane was distilled before used as mobile phase. Reagents and solvents were obtained from commercial sources and used without further purification. Dry toluene and THF were obtained from a solvent purification system PS-M6-6/7 from inert technologies. All sensitive reactions were carried out in dry glassware under nitrogen atmosphere.

HPLC was carried out on a JAI LC-9105 instrument with a Trentec Reprosil-100 Chiral-NR 8µm column using a 4:1 (V:V) mixture of *n*-hexane:dichloromethane as eluent to separate the enantiomers of **1**. Enantiomeric excess was determined to be >98% by HPLC trace integration.

UV/Vis absorption spectroscopy was measured on a Jasco V-670 spectrophotometer with 1 cm Hellma quartz glass cuvettes and spectroscopy grade solvents at room temperature.

Fluorescence spectra were recorded on a FLS980 from Edinburgh Instruments with 1 cm Hellma quartz glass cuvettes. Quantum yields were measured relative to *N,N′*-bis(2,6-diisopropylphenyl)-1,6,7,12-tetraphenoxy-3,4,9,10-perylenetetracarboxylic diimide (Φ = 0.96 in $CHCl_3$)[1] and calculated at four different excitation wavelengths. Time-resolved measurements were performed with an Edinburgh S3 Instruments picosecond pulsed laser diode (479.7 nm) in conjunction with a TCSPC detection unit.

CD/CPL spectra were measured on a Jasco CPL-300/J-1500 hybrid spectrometer with 1 cm Hellma quartz glass cuvettes and spectroscopy grade solvent at room temperature.

NMR spectroscopy was measured on Bruker Avance III HD 400 spectrometers. Chemical shifts are given in ppm and calibrated with regard to the residual solvent signal of $CDCl_3$ ($\delta(^1H)$ = 7.26 ppm, $\delta(^{13}C)$ = 77.16 ppm).[2] The multiplicities are given as abbreviations (s = singlet, d = doublet, t = triplet, m = multiplet), coupling constants (*J*) are given in Hertz (Hz).

Mass spectra were measured on a Bruker Daltonics ultrafleXtreme mass spectrometer (matrix-assisted laser desorption/ionisation time-of-flight, MALDI-TOF) in positive mode using *trans*-2-[3-(4-*tert*-butylphenyl)-2-methyl-2-propenylidene]malononitrile (DCTB) as matrix.

Cyclic and differential pulse voltammetry were measured with a standard commercial electrochemical analyser (EC epsilon; BAS Instruments, UK) with a three-electrode single-compartment cell. Tetrabutylammonium hexafluorophosphate ($(n\text{-Bu})_4NPF_6$) was applied as supporting electrolyte with ferrocene (Fc) as an internal standard for the calibration of potentials, Ag/AgCl as reference electrode, Pt disc and Pt wire as working and auxiliary electrodes, respectively. Cyclic voltammetry (CV) and differential pulse voltammetry (DPV) were performed at a scan rate of 100 mV/s at room temperature under argon atmosphere.

Melting points were measured with a polarization microscope BX41 from Olympus in conjunction with a temperature control element TP-94 by Linkam. All given melting points are uncorrected.

Differential scanning calorimetry (DSC) was performed on a DSC Q1000 with a DSC refrigerated cooling system (TA instrument Inc., USA) in aluminum Pans and Covers with a ramp speed of 10 K/s.

Single crystal X-ray diffraction data were collected at the P11 beamline at DESY by a single 360° scan φ sweep at 100 K. The diffraction data were indexed, integrated, and scaled using the XDS program package.[3] The structures were solved using SHELXT,[4] expanded with Fourier techniques and refined using the SHELX software package.[5] Hydrogen atoms were assigned at idealized positions and were included in the calculation of structure factors. All non-hydrogen atoms in the main residue were refined anisotropically. For the cocrystal of **1** and **6**, the diffraction was weak due to small crystal size. Thus, the resolution of measured diffraction data was until 1.2 Å (I/σ > 2.0). For the analysis of this data set enhanced rigid bond restraints (RIGU) were applied to the whole molecule to make ellipsoids reasonable. The checkcif routine[6] generated two level A alerts that are due to the low resolution of this data set. Absolute configuration of enantiopure crystal **(M)-1** was not assigned by crystallography but by CD spectra and TD-DFT calculations (Flack parameter was too low for assignment).

Theoretical calculations: Geometry optimizations, $NICS(1)_{zz}$ values, ACID and excited state calculations were performed using (time-dependent) density functional theory (DFT) at the level of B3LYP/6-311+G(d,p) as implemented in the Gaussian 09 program[7] unless otherwise stated. For the calculation of the dummy atoms' coordinates for $NICS(1)_{zz}$ and analysis of obtained data the multiwfn package was employed.[8] ALMO-EDA calculations[9] were done using the QChem 5.1 package.[10] The NCI surface was calculated with NCIPLOT 4.2[11] and visualized with vmd 1.9.3.[12]

2. Experimental procedures

1-Bromoanthracene

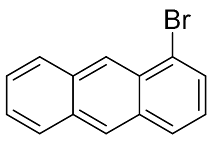

3

1-Bromoanthracene was synthesized by adapted literature procedures.[13]
To a solution of copper(II)bromide (25.0 g, 112 mmol, 1.25 equiv.) and tert-butyl nitrite (17.9 mL, 90%, 15.4 g, 134 mmol, 1.5 equiv.) in acetonitrile (400 mL) heated to 65 °C was added 1-aminoanthracene-9,10-dione (21.1 g, 95%, 89.6 mmol, 1.00 equiv.) as solid over the course of one hour. The mixture was then stirred for 3 h at 90 °C. Afterwards it was cooled to room temperature and diluted with 1 L of 1 M hydrochloric acid. The orange precipitate was filtered and washed with 200 mL of 1 M hydrochloric acid, 500 mL of water and 250 mL of ethanol. After drying under vacuum for one hour, the solid was further purified by silica column filtration (eluent: dichloromethane) yielding 25.0 g (87.1 mmol, 97%) of 1-bromoanthracene-9,10-dione as orange solids. These solids were suspended in 300 mL of isopropanol and cooled to 0 °C. Sodium borohydride (7.41 g, 196 mmol, 2.25 equiv.) was then added in one portion and stirring continued at 0 °C for 3 h. Afterwards the black suspension was heated to reflux for 15 min before recooling to 0 °C. To quench remaining borohydride 50 mL of 2 M hydrochloric acid were carefully and slowly added. The resulting mixture was then concentrated *in vacuo* to 75 mL, 250 mL of water were added and extracted with toluene (3 × 250 mL). The organic phases were dried over sodium sulfate and 26.5 g of a brown residue was obtained. This residue was then dissolved in 900 mL glacial acetic acid and tin(II)chloride (39.5 g, 209 mmol, 2.40 equiv.) was added in one portion. The mixture was then stirred at 100 °C for 2 h before cooling to room temperature and addition of 1 L of water, whereupon precipitation occurred. The solids and the aqueous phase were extracted with toluene (3 × 500 mL). The organic phases were dried over sodium sulfate. The resultant brown solid was then purified by silica-gel column chromatography (dry loaded on Celite, gradient elution cyclohexane to 10% dichloromethane in cyclohexane by volume).

Yield: 12.5 g (48.6 mmol, 54% over three steps) yellow solid. **^{1}H NMR** (400 MHz, $CDCl_3$, 298 K) *δ* (ppm) = 8.82 (s, 1H), 8.44 (s, 1H), 8.12–8.08 (m, 1H), 8.05–8.01 (m, 1H), 8.00–7.97 (m, 1H), 7.79 (dd, *J* = 7.1, 1.0 Hz, 1H), 7.54–7.51 (m, 2H), 7.30 (dd, *J* = 8.5, 7.1 Hz, 1H). The obtained ^{1}H NMR spectra were in accordance with previous reports.

1,1'-(2-Iodo-1,3-phenylene)dianthracene

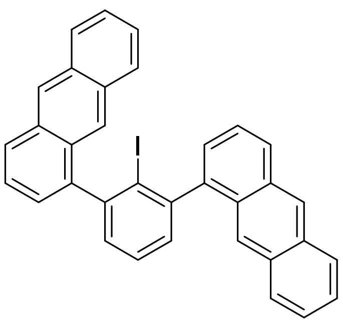

5

To a solution of 1,3-dichlorobenzene (200 µL, 1.75 mmol, 1.00 equiv.) in dry THF (2 mL) was added slowly a solution of *n*-buthyllithium in hexanes (1.31 mL, 1.6 M, 2.10 mmol, 1.20 equiv.) at −78 °C under nitrogen atmosphere and stirred for 2 h. In another vessel, freshly ground magnesium turnings (212 mg, 8.76 mmol, 5.00 equiv.) were overlayed with dry THF (2 mL) under nitrogen atmosphere. A solution of 1-bromoanthracene (1.80 g, 7.01 mmol, 4.00 equiv.) in dry THF (6 mL) was then prepared and one third was added by syringe. After the onset of Grignard formation (temperature increase and darkening color of solution towards black) the remaining bromide solution was then added slowly by syringe. Following complete addition the mixture was stirred for further 90 min at room temperature. Unreacted magnesium was discarded by cannula filtration, rinsed with dry THF (6 mL) and the dissolved Grignard reagent added to the colorless suspension of the organolithium species at –78 °C. The reaction mixture was then allowed to thaw to room temperature and stirred at 80 °C for 36 h. It was then cooled to 0 °C and iodine (1.78 g, 7.01 mmol, 4.00 equiv.) was added at once. After stirring at room temperature for 30 min, the reaction was quenched by addition of saturated aqueous sodium sulfite (20 mL). Ethyl acetate (75 mL) and additional water (50 mL) were added, the phases were separated and the aqueous extracted twice more with ethyl acetate (2 × 75 mL). The combined organic phases were washed with brine (100 mL) and then dried over sodium sulfate. The crude brown solid was then purified by silica-gel column chromatography (dry loaded on Celite, gradient elution cyclohexane to 10% dichloromethane in cyclohexane by volume).

Yield: 223.0 mg (401 µmol, 23%) pale yellow solid. **^{1}H NMR** (400 MHz, $CDCl_3$, 298 K) *δ* (ppm) = 8.52 (s, 2H), 8.23 (s, 1H), 8.13–8.09 (m, 3H), 8.05–8.00 (m, 3H), 7.96–7.92 (m, 1H), 7.71–7.65 (m, 1H), 7.60–7.43 (m, 10H).**^{13}C{^{1}H} NMR** (101 MHz, CD_2Cl_2, 298 K) δ (ppm) =. 146.9, 146.8, 143.3 (2×), 131.9 (2×), 131.8, 131.7, 130.3, 130.2, 130.0, 128.8, 128.7, 128.6, 128.2 (2×), 128.1, 127.9, 126.9 (2×), 126.8, 126.5, 125.8, 125.7, 125.5, 125.1, 125.0 (2×), 124.9. **HRMS** (MALDI-TOF): *m/z*: calculated for $[C_{32}H_{21}I]^+$: 556.06824, *m/z* found: 556.06848. **Melting point**: 265–266 °C.

19c-boratribenzo[*gh*,*jk*,*mn*][6]helicene

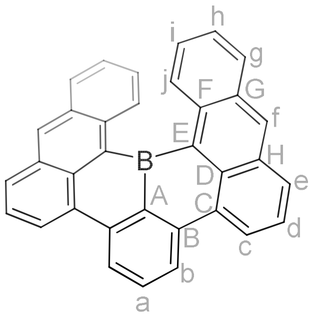

1

To a suspension of **5** (30.0 mg, 53.9 µmol, 1.00 equiv.) in dry toluene (1.5 mL) was slowly added *n*-buthyllithium in hexanes (67 µL, 1.6 M, 0.11 mmol, 2.0 equiv.) at 0 °C under nitrogen atmosphere. The mixture was then stirred for 90 min at 0 °C. To the cream-colored suspension was then dropwise added a solution of boron tribromide in dichloromethane (0.11 mL, 1.0 M, 0.11 mmol, 2.0 equiv.) at 0 °C. After stirring at 0 °C for 1 h the orange-brown suspension was heated to 50 °C for 1 h. To the resultant orange solution DIPEA in dry toluene (0.22 mL, 0.50 M, 0.11 mmol, 2.0 equiv.) was added at 0 °C. Afterwards the reaction mixture was stirred at 100 °C for 18 h. The red, intensely orange fluorescent solution was then cooled to room temperature, diluted with toluene and filtered through a silica gel plug (eluent toluene). The crude red solid was then further purified by silica gel column chromatography (dry loaded onto Celite, eluent cyclohexane:dichloromethane 9:1 by volume) and subsequent washing with *n*-hexane.

Yield: 4.0 mg (9.1 µmol, 17%) purple solid. **^{1}H NMR** (400 MHz, $CDCl_3$, 298 K) δ (ppm) = 8.81 (s, 2H, H_f), 8.70 (dd, J = 7.2, 1.0 Hz, 2H, H_c), 8.45 (d, J = 8.0 Hz, 2H, H_b), 8.25 (d, J = 7.9 Hz, 2H, H_e), 8.02 (td, J = 8.4, 0.6 Hz, 2H, H_g), 7.84 (t, J = 7.9 Hz, 1H, H_a), 7.77 (dd, J = 8.3, 7.2 Hz, 2H, H_d), 7.64 (dd, J = 8.8, 0.8 Hz, 2H, H_j), 7.31 (ddd, J = 8.3, 6.6, 1.1 Hz, 2H, H_h), 6.75 (ddd, J = 8.8, 6.6, 1.3 Hz, 2H, H_i). **^{13}C{^{1}H} NMR** (101 MHz, $CDCl_3$, 298 K): δ (ppm) =.138.4 (C_B), 138.1 (C_F), 134.7 (C_f), 133.5 (C_C), 131.8 (C_D), 131.6 (C_G), 131.3 (C_H), 131.0 (C_a), 130.9 (C_e), 130.7 (C_j), 128.5 (C_g), 126.3 (C_i), 125.6, (C_h)125.3 (2×, C_c & C_d), 122.6 (C_b). No ^{13}C signals in α-position to the boron center were observed (C_A & C_E). **^{11}B{^{1}H} NMR** (128 MHz, $CDCl_3$, 298 K): Not observed due to low solubility. **HRMS** (MALDI-TOF): *m*/*z* calculated for $[C_{34}H_{19}B]^+$: 438.15743, *m*/*z* found: 438.15739. **UV/Vis** ($CHCl_3$, 298 K): λ_{max} = 560 nm (ε = 16200 L mol^{-1} cm^{-1}), 525 nm (ε = 9400 L mol^{-1} cm^{-1}), 430 nm (ε = 7000 L mol^{-1} cm^{-1}). **Fluorescence** ($CHCl_3$, 298 K, λ_{exc} = 525 nm): λ_{max} = 587 nm (Φ = 0.70, τ = 9.5 ns, Stokes' shift 820 cm^{-1}). **Cyclic voltammetry** (c ≈ 2.5×10^{-4} M, 0.1 M *n*-Bu_4NPF_6 in CH_2Cl_2 at 298 K, E_{redox} vs. Fc^+/Fc): E_{red1} = −1.58 V, E_{ox1} = 0.66 V, E_{ox2}= 0.96 V. **Melting point**: >350 °C.

3. NMR spectra

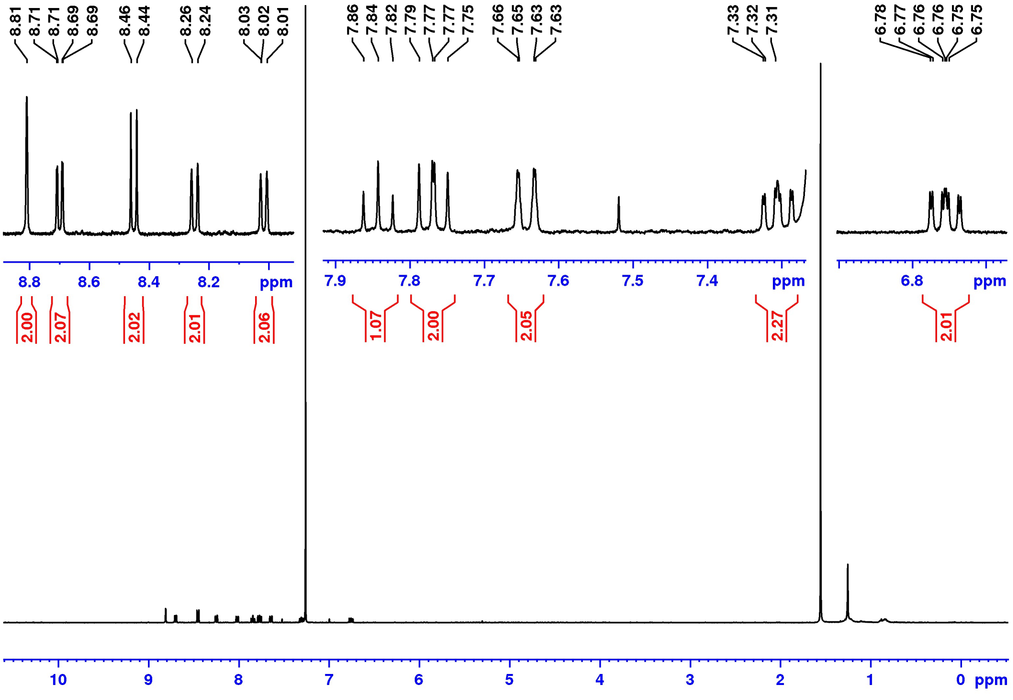

Figure S1. ^{1}H NMR (400 MHz, 298 K) spectrum of **1** in $CDCl_3$.

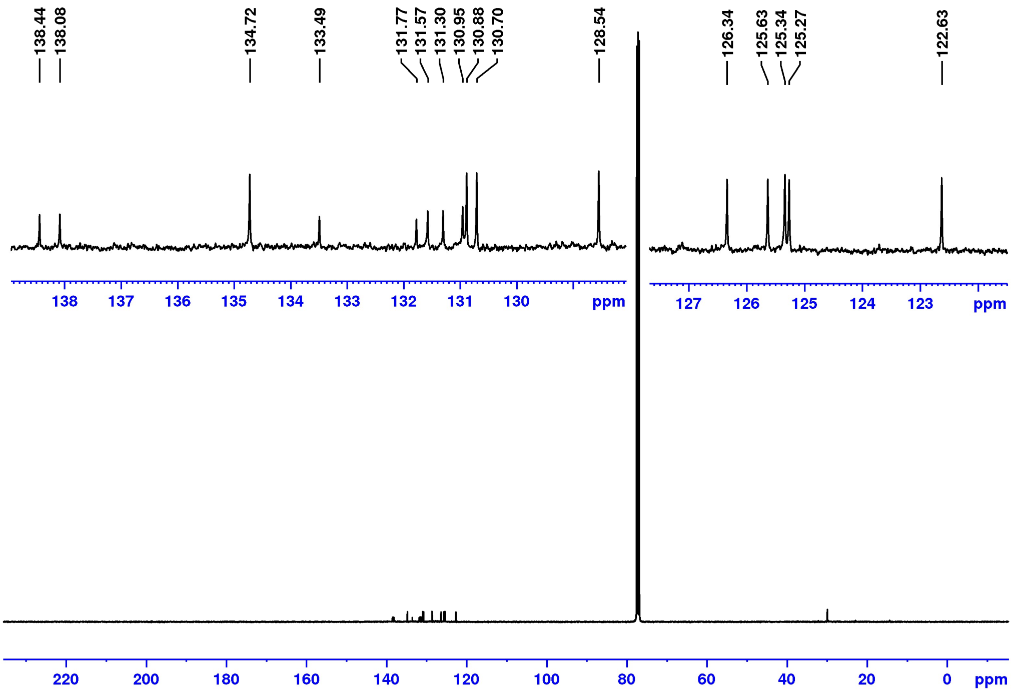

Figure S2. ^{13}C NMR (101 MHz, 298 K) spectrum of **1** in $CDCl_3$.

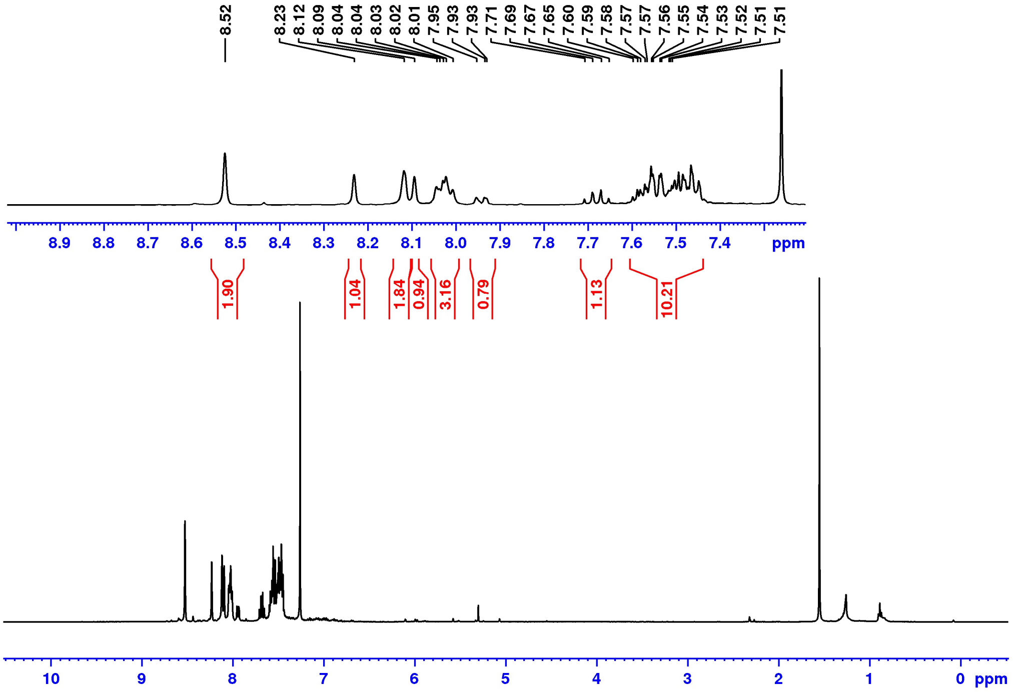

Figure S3. ^{1}H NMR (400 MHz, 298 K) spectrum of **5** in $CDCl_3$.

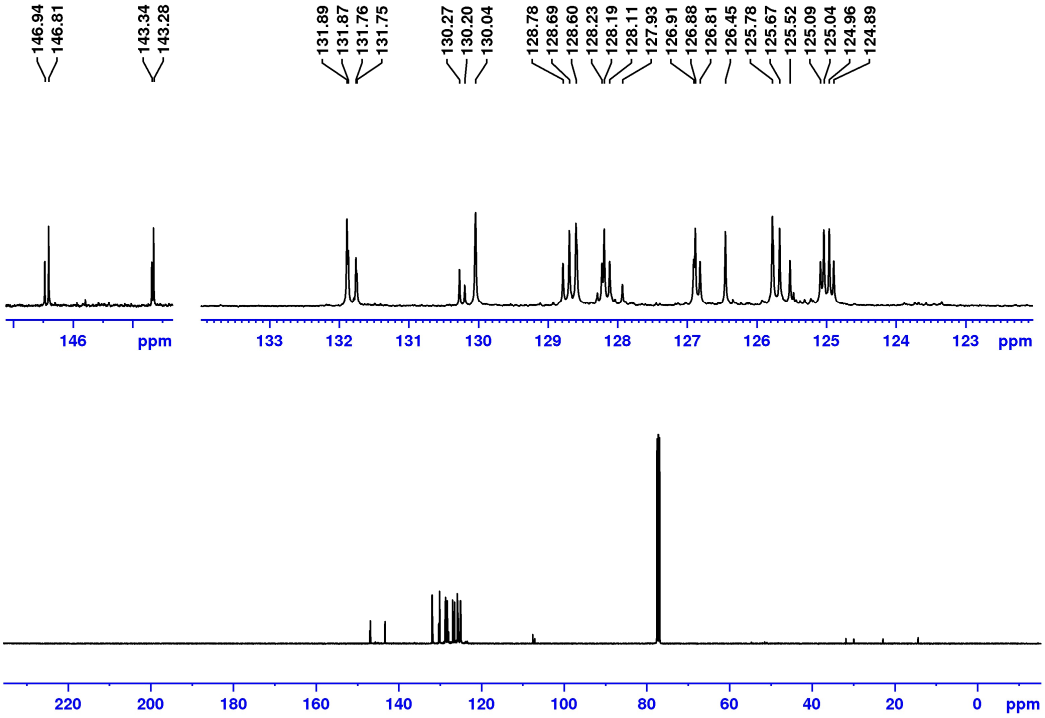

Figure S4. ^{13}C NMR (101 MHz, 298 K) spectrum of **5** in $CDCl_3$.

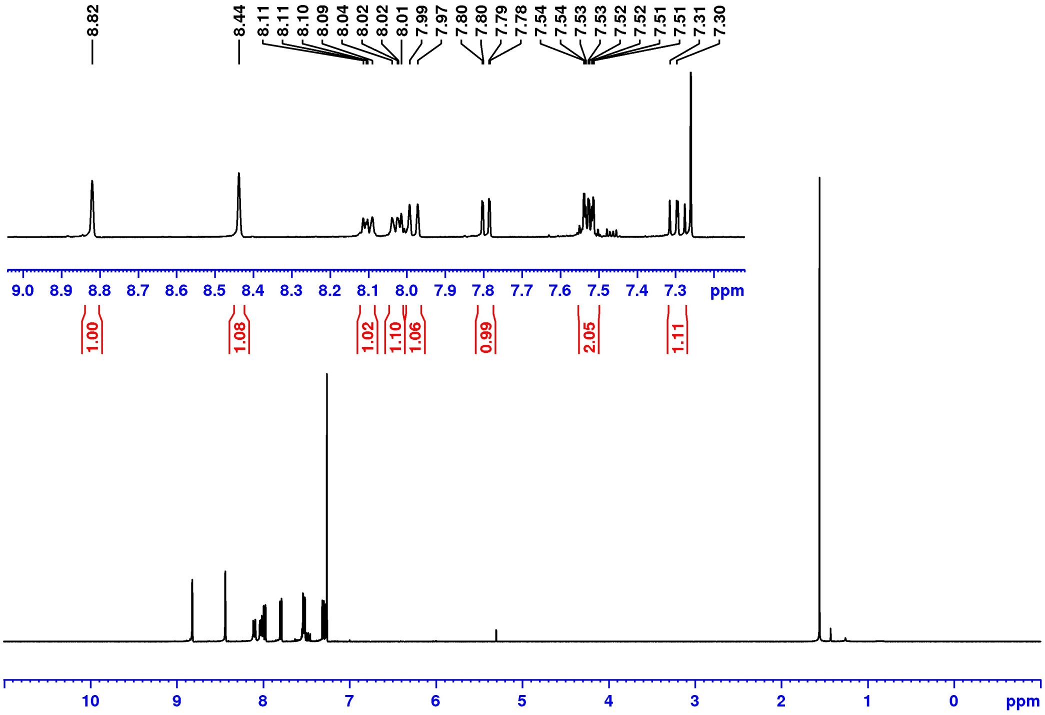

Figure S5. ^{1}H NMR (400 MHz, 298 K) spectrum of **3** in $CDCl_3$.

4. Cyclic voltammetry

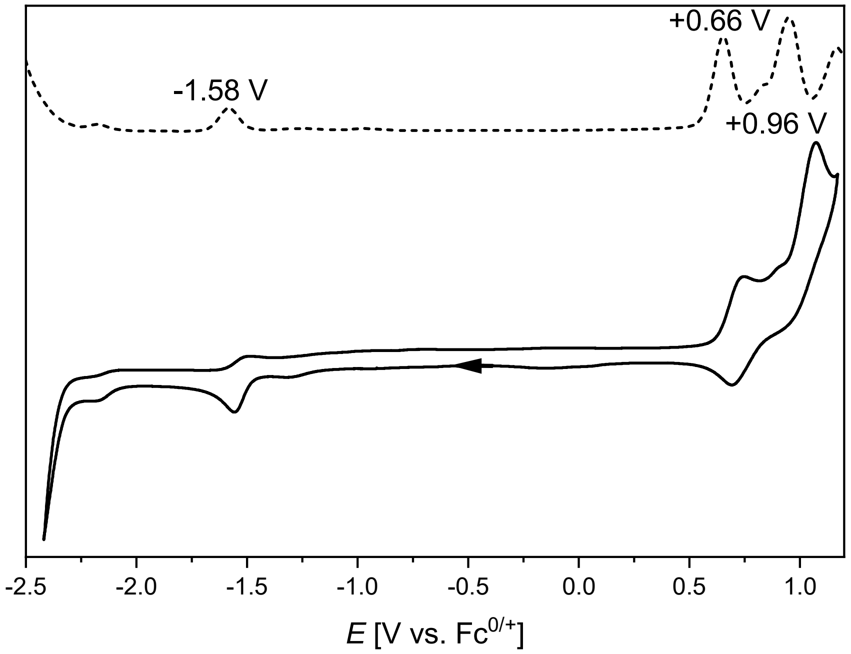

Figure S6. Cyclic voltammogram (solid line) and differential pulse voltammogram (dashed line) of **1** taken at 298 K in dry, degassed dichloromethane ($c \approx 2.5 \times 10^{-4}$ M) with 0.1 M (n-Bu)$_4$NPF$_6$ under argon atmosphere at a scan rate of 100 mV/s. Scan direction shown as arrow.

5. UV/Vis absorption and fluorescence spectra

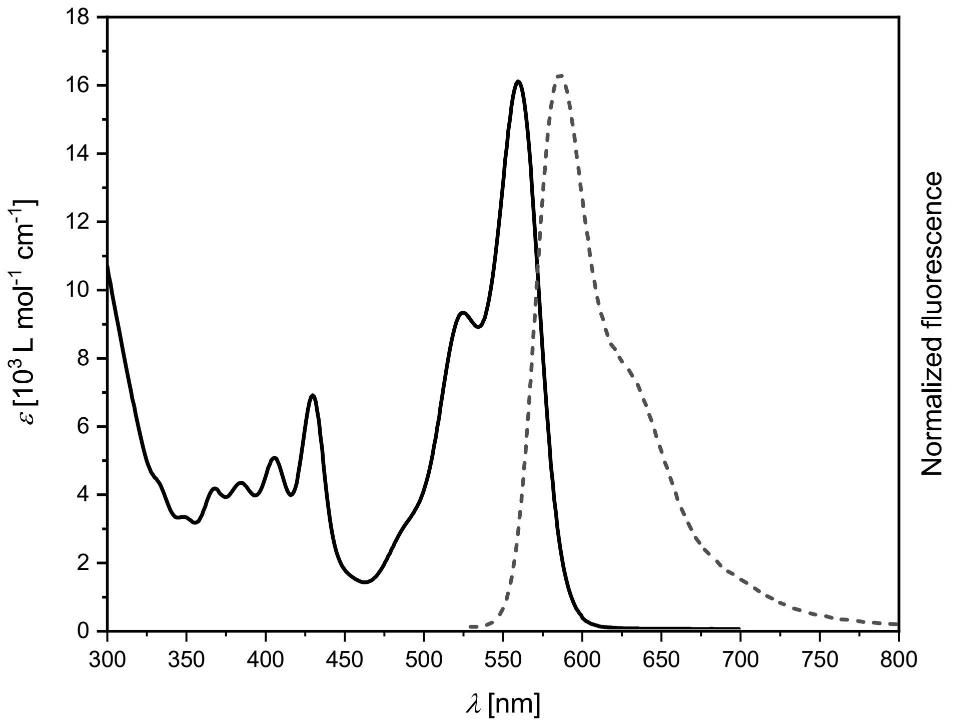

Figure S7. UV/Vis absorption (solid line) and fluorescence spectrum (dashed line) of **1** in chloroform at 298 K (c = 1.86×10^{-5} M for UV/Vis, diluted to OD ≈ 0.05 for fluorescence, λ_{exc} = 525 nm).

Table S1. Selected emission properties of bora[6]helicene **1** in different solvent systems at 298 K.

	$\Phi \pm s$ [b]	$\tau \pm s$ [c]	λ_{abs}	λ_{em}	*Stokes' shift*
$CHCl_3$ (ambient)	0.70 ± 0.01	9.5 ns ± 0.02 ns	560 nm	587 nm	800 cm^{-1}
(degassed)[a]	0.77 ± 0.02	10.1 ns ± 0.01 ns			
CCl_4 (ambient)	0.80 ± 0.03	9.3 ns ± 0.01 ns	560 nm	580 nm	600 cm^{-1}
(degassed)[a]	0.81 ± 0.03	9.8 ns ± 0.01 ns			
PhMe (ambient)	0.82 ± 0.03	9.1 ns ± 0.01 ns	563 nm	583 nm	600 cm^{-1}
(degassed)[a]	0.84 ± 0.03	9.7 ns ± 0.01 ns			

a) Solvents were degassed by purging with nitrogen for 20 min. b) Quantum yields were measured relative to *N,N'*-bis(2,6-diisopropylphenyl)-1,6,7,12-tetraphenoxy-3,4,9,10-perylenetetracarboxylic diimide (Φ = 0.96 in $CHCl_3$)[1] and calculated at four different excitation wavelengths (515, 520, 525, 530 nm). They are given as averages of the four wavelengths with their respective standard deviation. c) Life times measured after excitation with a pulsed 479.7 nm picosecond laser and given with the standard deviation of the fit.

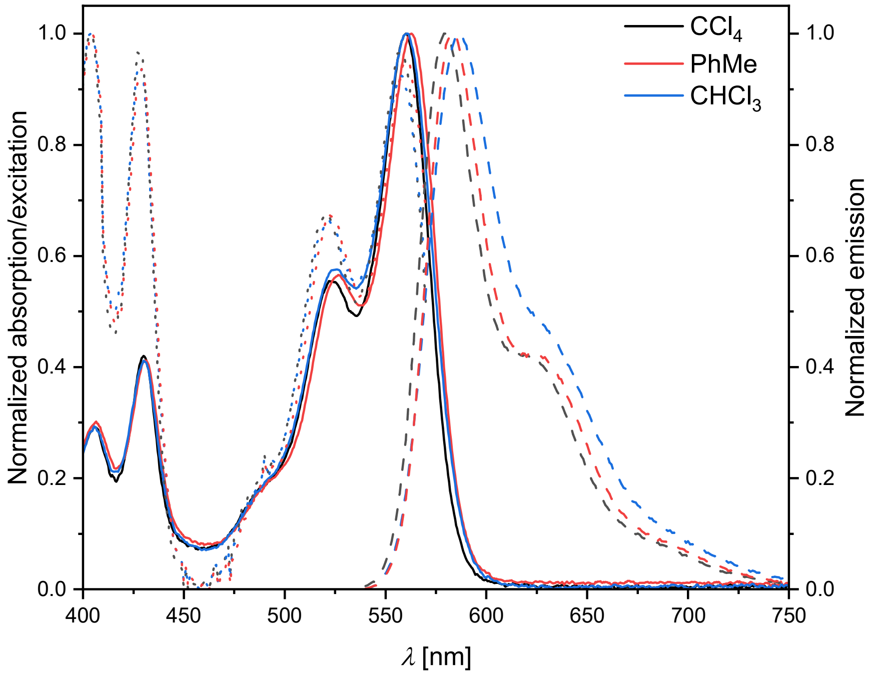

Figure S8. UV/Vis absorption (solid lines), excitation (dotted lines, λ_{obs} = 587 nm for $CHCl_3$, λ_{obs} = 580 nm for CCl_4 and PhMe) and emission (dashed lines, λ_{exc} = 520 nm) spectra of **1** in different solvents (OD ≈ 0.05) measured at 298 K. Identical spectra were obtained for aerated and degassed solutions.

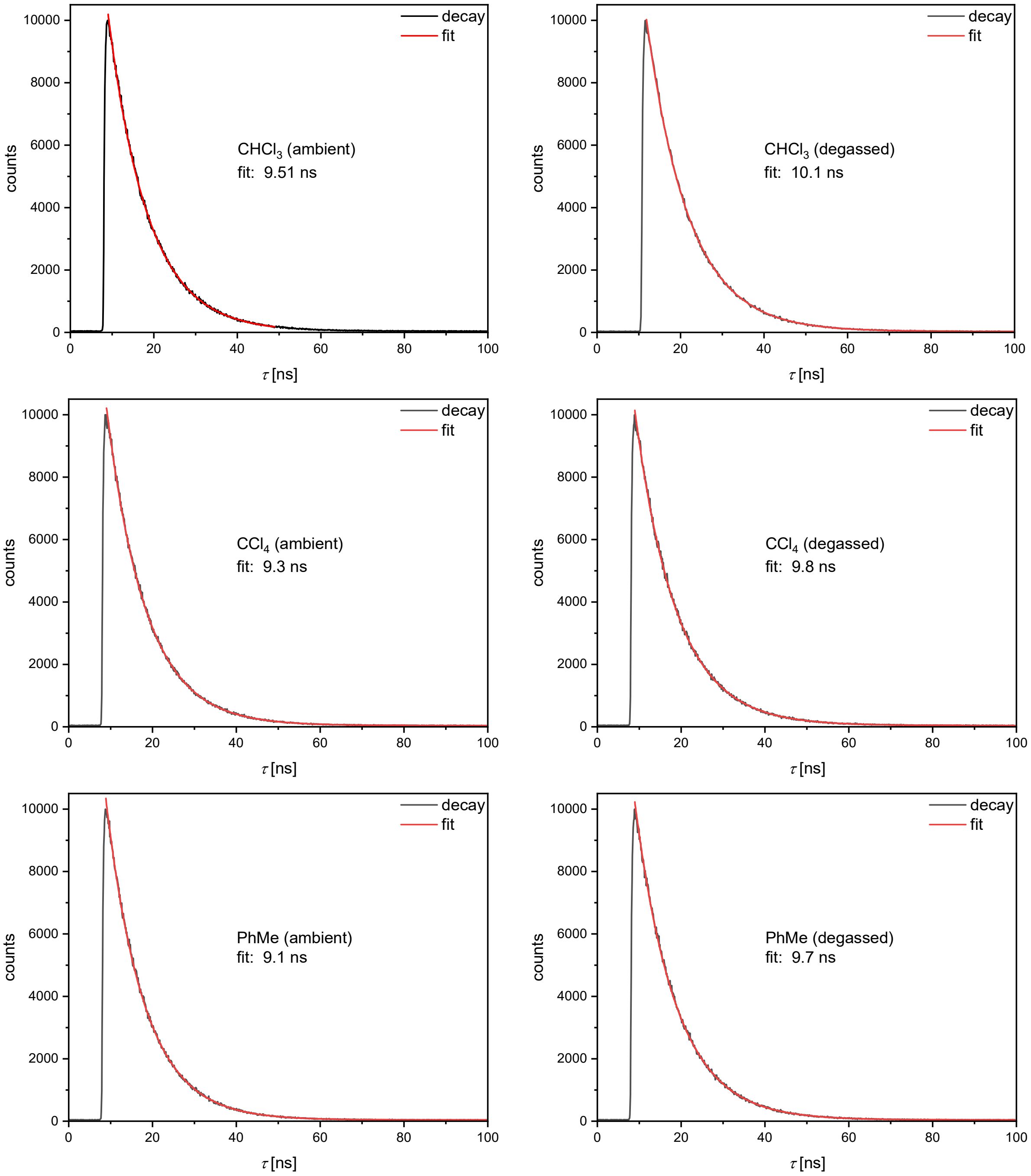

Figure S9. Fluorescence decay of **1** in various solvents (OD ≈ 0.05) with fit after excitation with a pulsed 479.7 nm picosecond laser at 298 K. Solvents were degassed by purging the samples with nitrogen for 20 minutes.

6. CD/CPL spectra

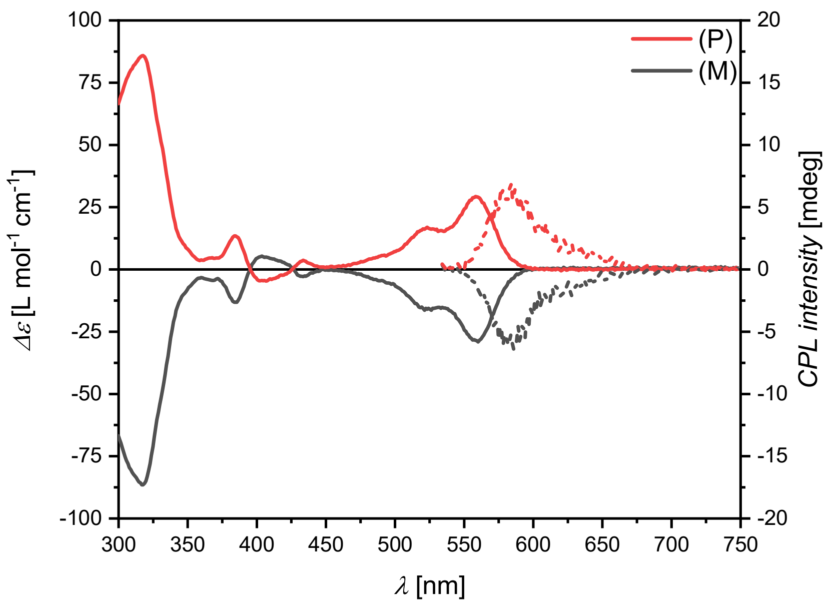

Figure S10. CD and CPL spectra of **1** in chloroform at 298 K (c = 5×10^{-5} M for CD, diluted to OD ≈ 0.1 for CPL, λ_{exc} = 430 nm).

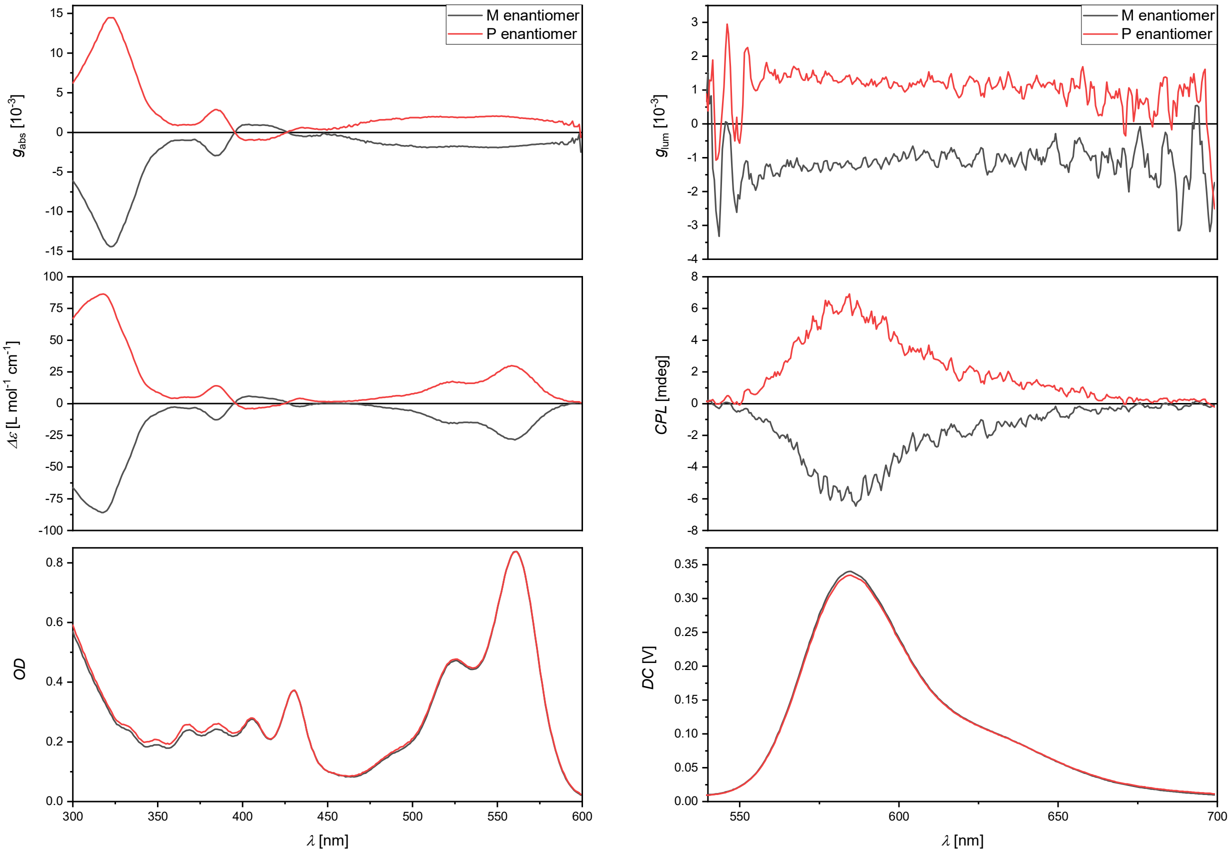

Figure S11. Plots of g_{abs}, $\Delta\varepsilon$, OD of CD measurements of **1** (left). Plots of g_{lum}, CPL raw data and DC detector voltage of **1** (right). All spectra taken in chloroform at 295 K (c = 5×10^{-5} M for CD, diluted to OD ≈ 0.1 for CPL, λ_{exc} = 430 nm).

7. MALDI-TOF-MS spectra

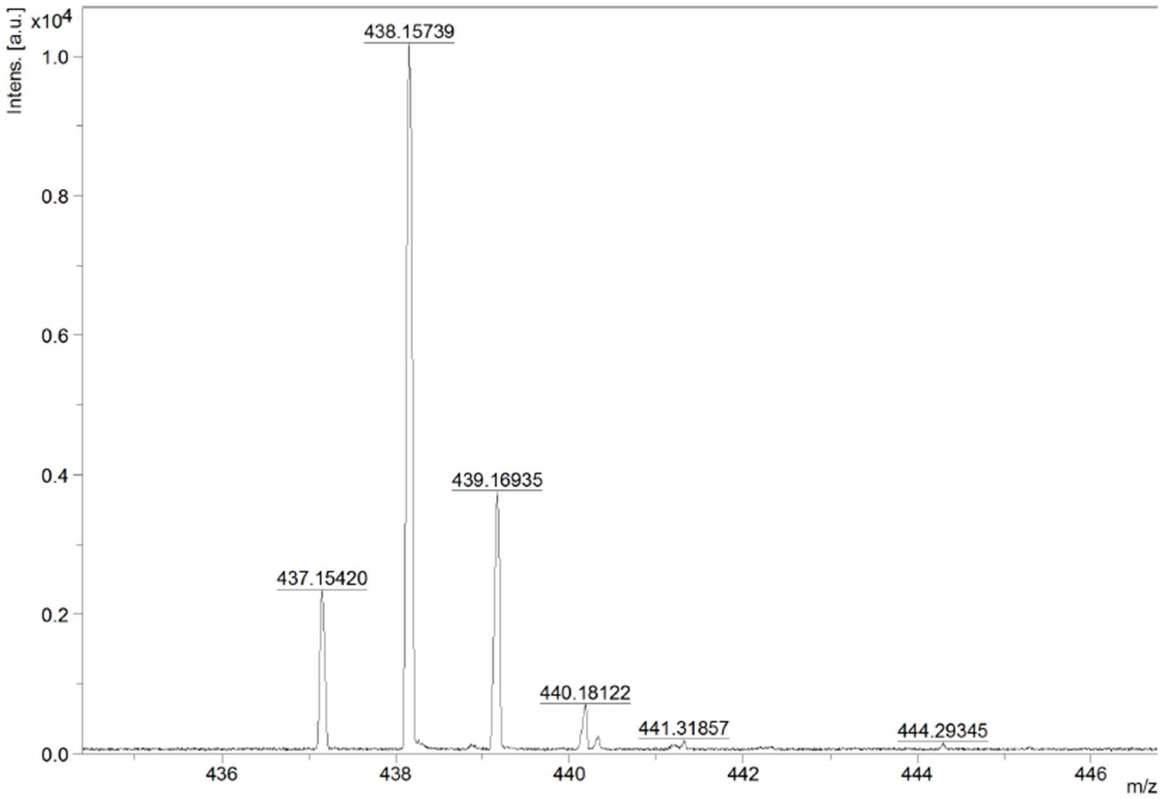

Figure S12. Isotope pattern of **1** in its MALDI-TOF-MS (DCTB, positive mode).

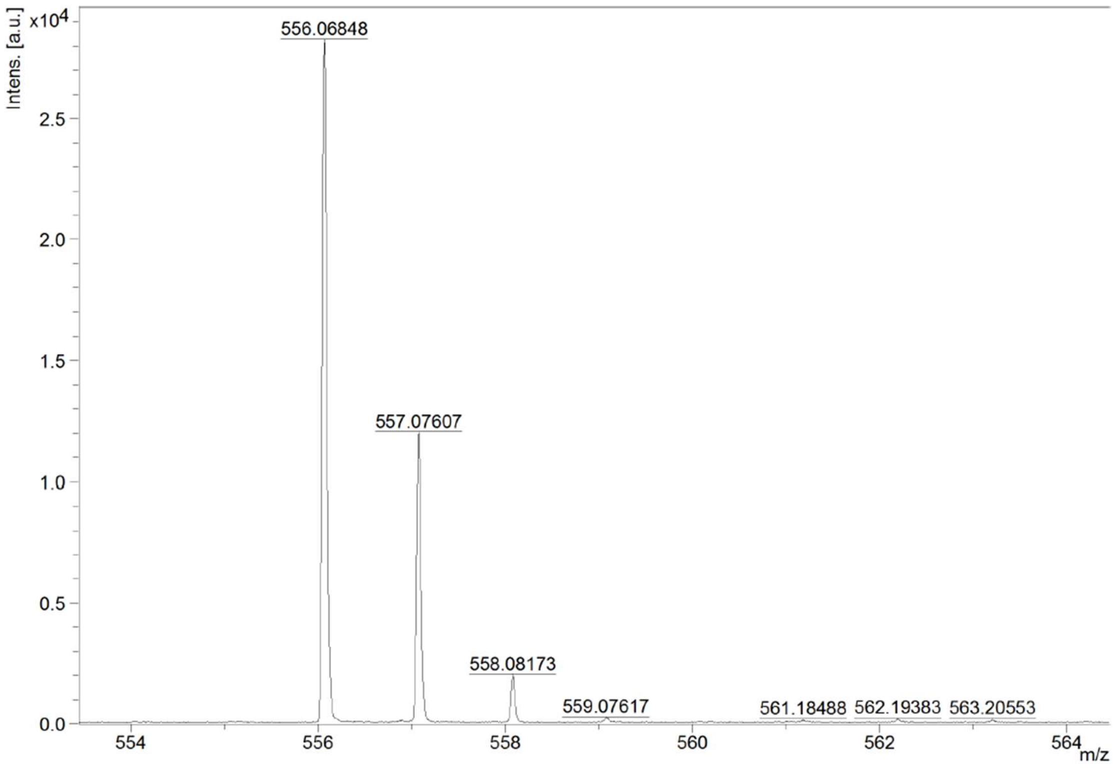

Figure S13. Isotope pattern of **5** in its MALDI-TOF-MS (DCTB, positive mode).

8. Chiral separation and racemization studies

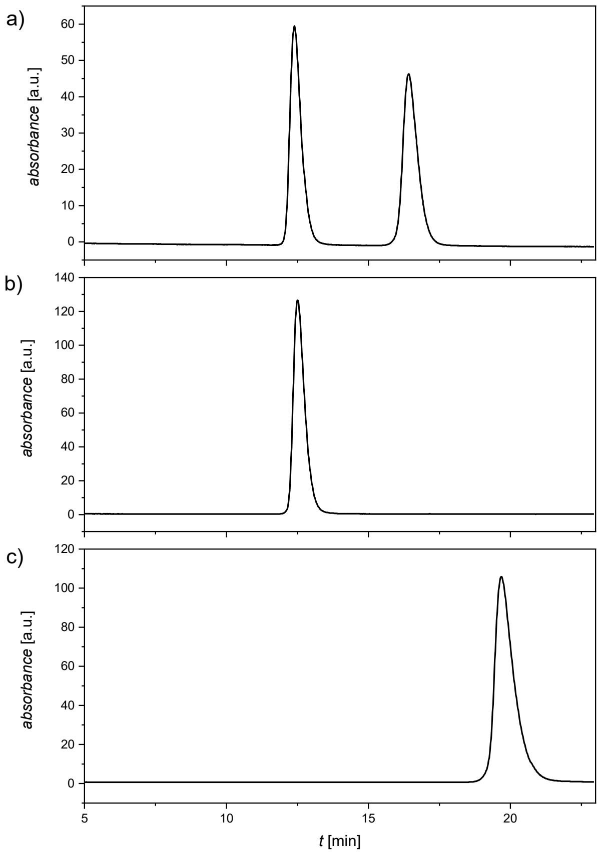

Figure S14. HPLC trace of a) racemate, b) (*P*) and (*M*) enantiomer of **1**, respectively. Detected at 554 nm.

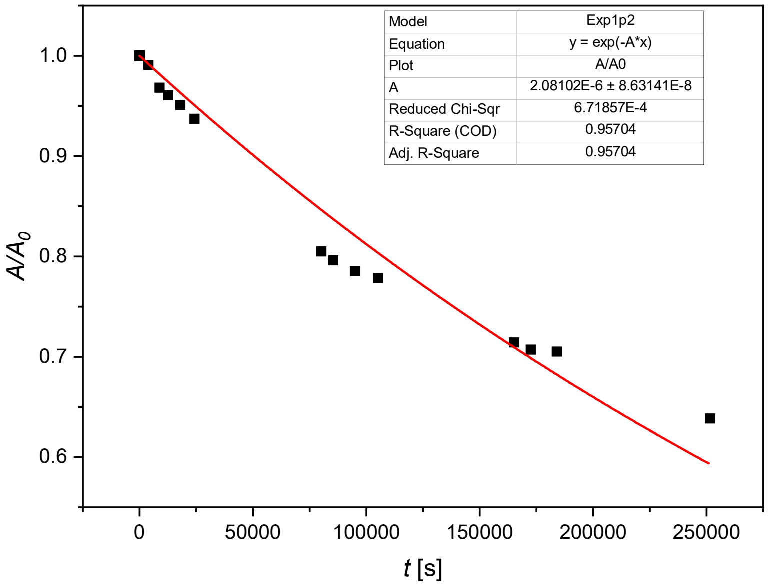

Figure S15. Racemization of **1** at 150 °C in *ortho*-dichlorobenzene as determined by integration of analytical HPLC plots with detection at 554 nm.

9. Stability measurements

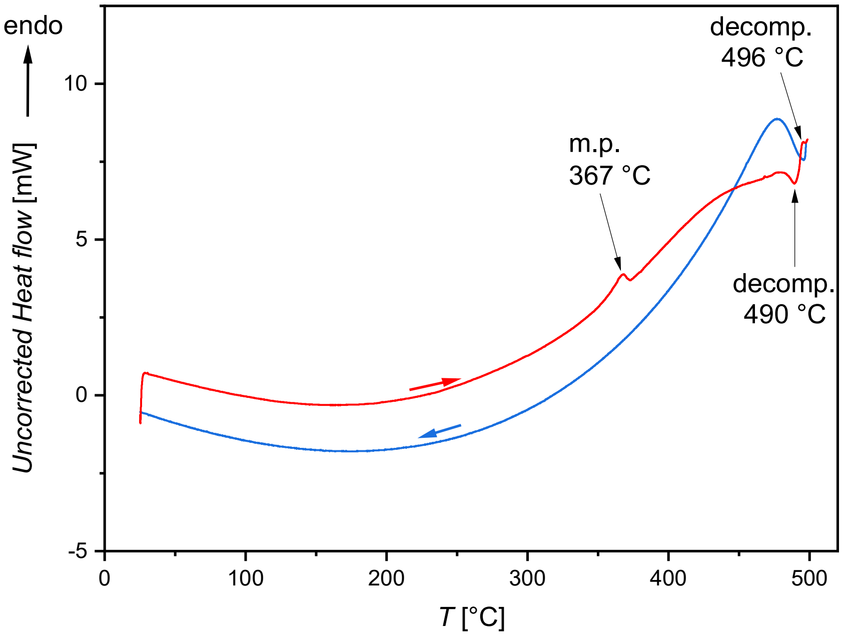

Figure S16: Differential scanning calorimetry measurement of **1** with a sample prepared under ambient conditions.

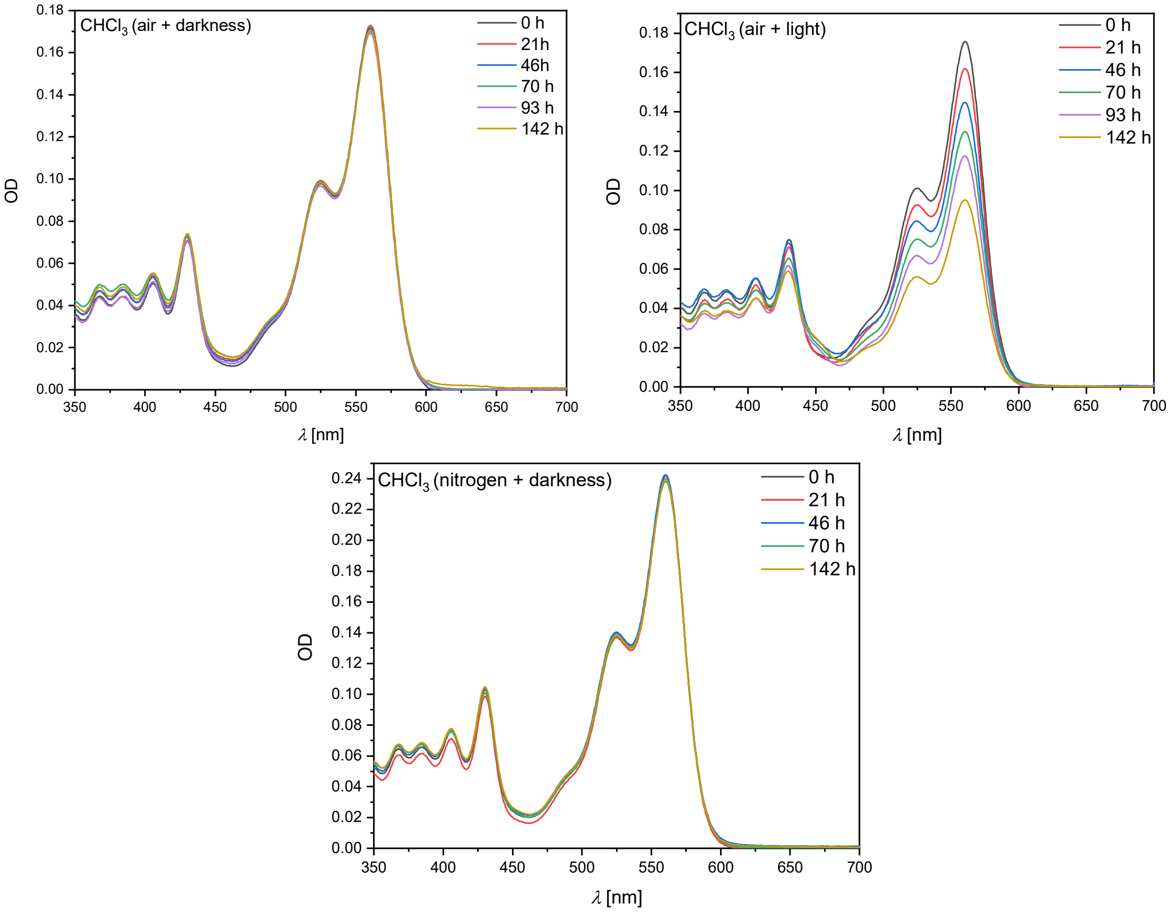

Figure S17: UV/Vis absorption spectra of **1** in chloroform (c ≈ 1×10^{-5} M) after storage under different conditions.

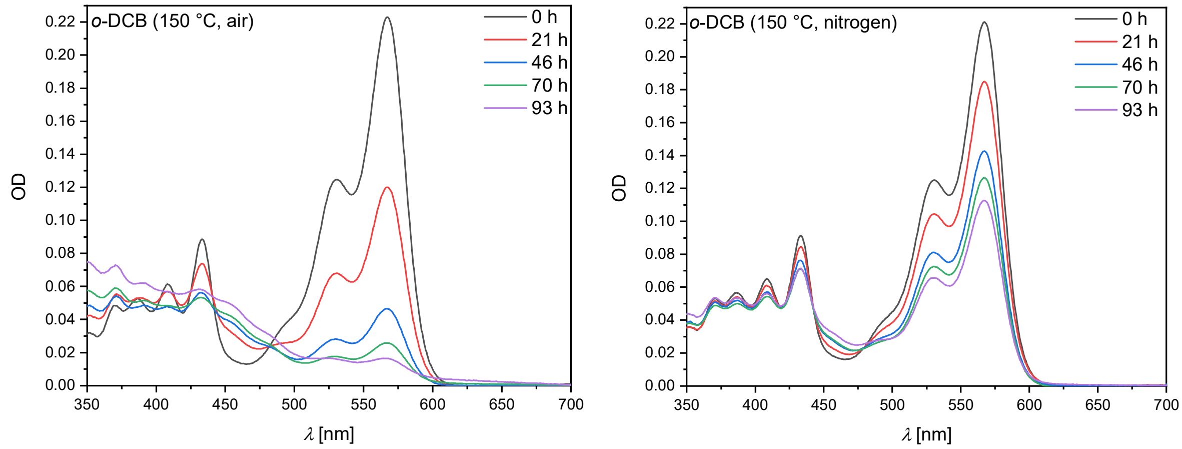

Figure S18: UV/Vis absorption spectra of **1** in ortho-dichlorobenzene (c ≈ 1×10^{-5} M) after heating to 150 °C (oil bath) under laboratory daylight.

10. Crystal structure data

Table S2. Crystallographic data for racemic **1**.

Empirical formula	$C_{34}H_{19}B$
Formula weight	438.30
Temperature	100(2) K
Wavelength	0.61992 Å
Crystal system	Monoclinic
Space group	*P2/c*
Unit cell dimensions	a = 20.6520(17) Å α = 90°<br>b = 4.9020(14) Å β = 109.110(11)°<br>c = 21.6450(14) Å γ = 90°
Volume	2070.5(6) Å^3
Z	4
Density (calculated)	1.406 g/cm^3
Absorption coefficient	0.061 mm^{-1}
F(000)	912
Crystal size	0.050 × 0.010 × 0.010 mm^3
Theta range for data collection	0.910 to 27.930°
Index ranges	$-26 \le h \le 26$, $-5 \le k \le 5$, $-24 \le l \le 27$
Reflections collected	5460
Independent reflections	5460 [*R*(int) = ?]
Completeness to theta = 21.836°	96.7%
Absorption correction	None
Refinement method	Full-matrix least-squares on F^2
Data / restraints / parameters	5460 / 0 / 318
Goodness-of-fit on F^2	1.164
Final *R* indices [$I > 2\text{sigma}(I)$]	R_1 = 0.0738, wR_2 = 0.2748
R indices (all data)	R_1 = 0.0794, wR_2 = 0.2818
Largest diff. peak and hole	0.441, –0.417 e.Å^{-3}

Table S3. Crystallographic data for ***(M)*-1**.

Empirical formula	$C_{34}H_{19}B$
Formula weight	438.30
Temperature	100(2) K
Wavelength	0.6199 Å
Crystal system	Trigonal
Space group	*R3*
Unit cell dimensions	*a* = 34.6300(19) Å α = 90°<br>*b* = 34.6300(19) Å β = 90°<br>*c* = 4.8030(4) Å γ = 120°
Volume	4988.2(7) Å^3
Z	9
Density (calculated)	1.313 g/cm^3
Absorption coefficient	0.057 mm^{-1}
F(000)	2052
Crystal size	0.100 × 0.010 × 0.010 mm^3
Theta range for data collection	1.026 to 27.811°
Index ranges	$-51 \leq h \leq 51$, $-44 \leq k \leq 44$, $-7 \leq l \leq 7$
Reflections collected	30905
Independent reflections	6301 [*R*(int) = 0.0369]
Completeness to theta = 21.836°	99.3%
Absorption correction	None
Refinement method	Full-matrix least-squares on F^2
Data / restraints / parameters	6301 / 1 / 316
Goodness-of-fit on F^2	1.068
Final *R* indices [*I* > 2sigma(*I*)]	R_1 = 0.0474, wR_2 = 0.1249
R indices (all data)	R_1 = 0.0523, wR_2 = 0.1291
Largest diff. peak and hole	0.177, –0.221 e.Å^{-3}

Table S4. Crystallographic data for **1•6** cocrystal.

Empirical formula	$C_{88}H_{64}BN_3O_4$
Formula weight	1238.23
Temperature	100(2) K
Wavelength	0.6199 Å
Crystal system	Monoclinic
Space group	*P2₁/c*
Unit cell dimensions	*a* = 26.86(2) Å α = 90°<br>*b* = 14.399(15) Å β = 106.99(11)°<br>*c* = 16.935(17) Å γ = 90°
Volume	6264(11) Å^3

Z	4
Density (calculated)	1.313 g/cm^3
Absorption coefficient	0.061 mm^{-1}
F(000)	2600
Crystal size	0.050 × 0.005 × 0.005 mm^3
Theta range for data collection	1.414 to 23.099°
Index ranges	$-33 \le h \le 31$, $-18 \le k \le 18$, $-21 \le l \le 21$
Reflections collected	91085
Independent reflections	12534 [R(int) = 0.2593]
Completeness to theta = 21.836°	96.2%
Absorption correction	None
Refinement method	Full-matrix least-squares on F^2
Data / restraints / parameters	12534 / 882 / 873
Goodness-of-fit on F^2	1.179
Final R indices [$I > 2\text{sigma}(I)$]	R_1 = 0.1427, wR_2 = 0.3528
R indices (all data)	R_1 = 0.3558, wR_2 = 0.4546
Largest diff. peak and hole	0.296, –0.247 e.Å^{-3}

11. Computational data

11.1. NICS and ACID data

Table S5. NICS$_{zz}$ values[14] of **1** calculated at the GIAO-B3LYP-6-311+G(d,p) level of theory. Due to non-planarity the values for NICS$_{zz}$(1) and NICS$_{zz}$(-1) differ and are averaged to yield NICS$_{zz}$(avg.).[15]

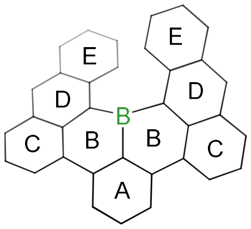

	A	B	C	D	E
NICS$_{zz}$(1)	−27.0	−28.5	−21.5	8.3	−19.2
NICS$_{zz}$(-1)	−24.9	−27.8	−20.2	8.1	−19.2
NICS$_{zz}$(avg)	−25.9	−28.1	−20.9	8.2	−19.2

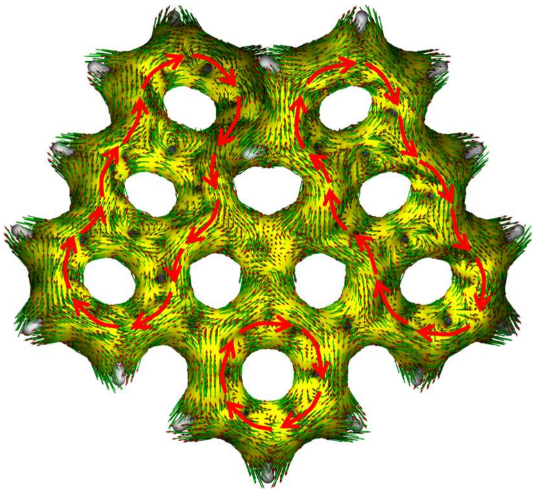

Figure S19. ACID plot[16] of **1** (isovalue 0.05) with paratropic (aromatic) ring currents marked in red. All calculations were performed at B3LYP/6-311+G(d,p) level of theory.

11.2. Frontier molecular orbitals

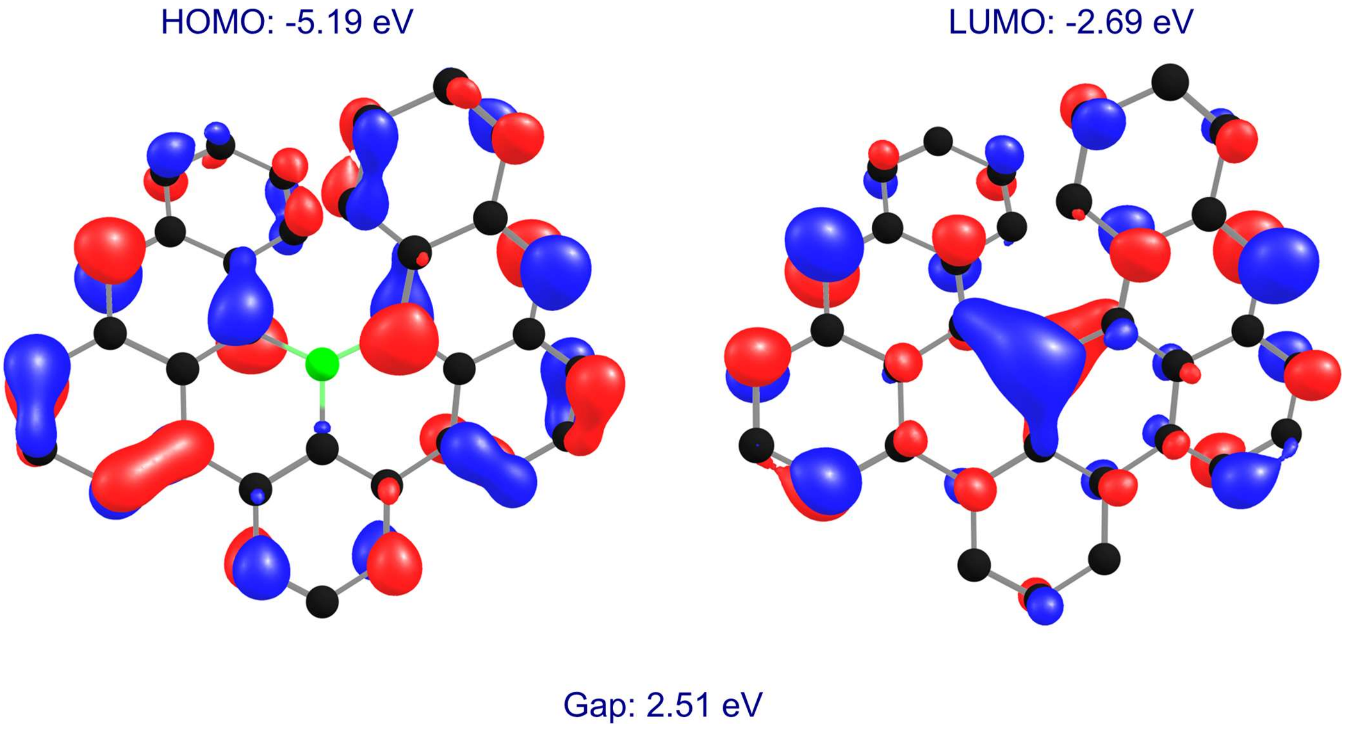

Figure S20. Frontier molecular orbitals calculated at the B3LYP/6-311+G(d,p) level of theory (iso-value 0.04).

11.3. TD-DFT data

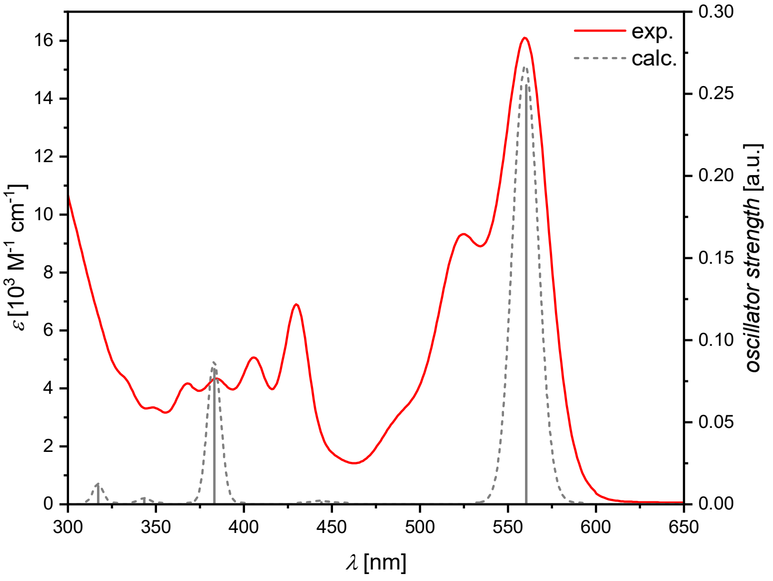

Figure S21. Experimental (solid line) and calculated UV/Vis spectra of **1**. TD-DFT calculations were performed at the B3LYP/6-311+G(d,p) level of theory with a 50/50 split of singlets and triplets calculated. The calculated spectra were shifted by 91 nm towards higher energies, scaled by a factor of 0.11 and convoluted with a phenomenological Gaussian with a FWHM of 0.05 eV.

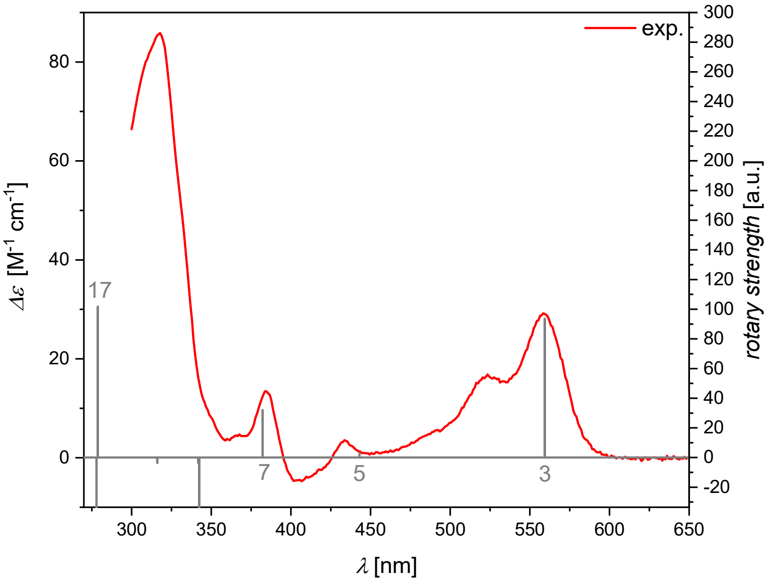

Figure S22. Experimental CD spectra of (*P*)-**1** and calculated transitions with their rotary strengths indicated in grey. TD-DFT calculations were performed at the B3LYP/6-311+G(d,p) level of theory with a 50/50 split of singlets and triplets calculated. The calculated transitions were shifted by 92 nm towards higher energies.

Table S6. Ground to excited state transition electric dipole moments (μ) and ground to excited state transition magnetic dipole moments (m) taken from TD-DFT calculations of (*P*)-**1** at the B3LYP/6-311G* level of theory with a 50/50 split of singlets and triplets calculated. The dissymmetry factors were calculated according to $g_{abs} = 4\cos(\theta)\,\frac{|\vec{\mu}||\vec{m}|}{|\vec{\mu}|^2+|\vec{m}|^2}$.

Excited state	$\|\mu\|$ [10^{-19} esu cm^{-1}]	$\|m\|$ [10^{-22} erg G^{-1}]	Θ [°]	g_{abs}(calc.) [10^{-3}]
3 (S_1)	59.5	92.2	99.6	3.82
5	3.71	6.23	180	4.02
9	28.8	10.5	180	0.87
17	10.4	128.5	143	19.4

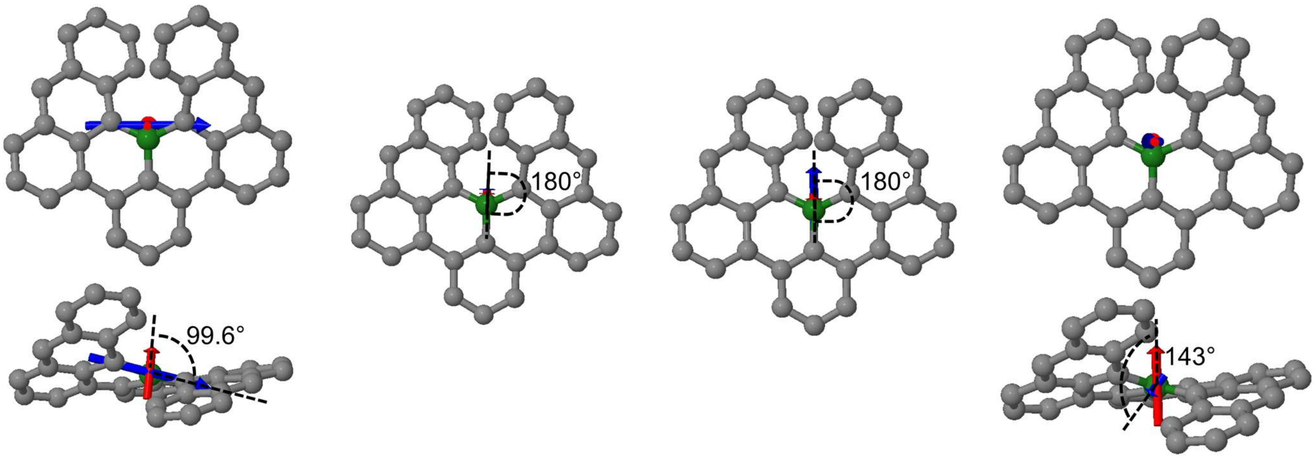

Figure S23. Graphical representation of magnetic (*m*, red) as well as electric (*μ*, blue) transition dipole moments of selected excited states of (*P*)-**1**. Claculations were performed at the B3LYP/6-311+G(d,p) level of theory with a 50/50 split of singlets and triplets.

11.4. Racemization barrier

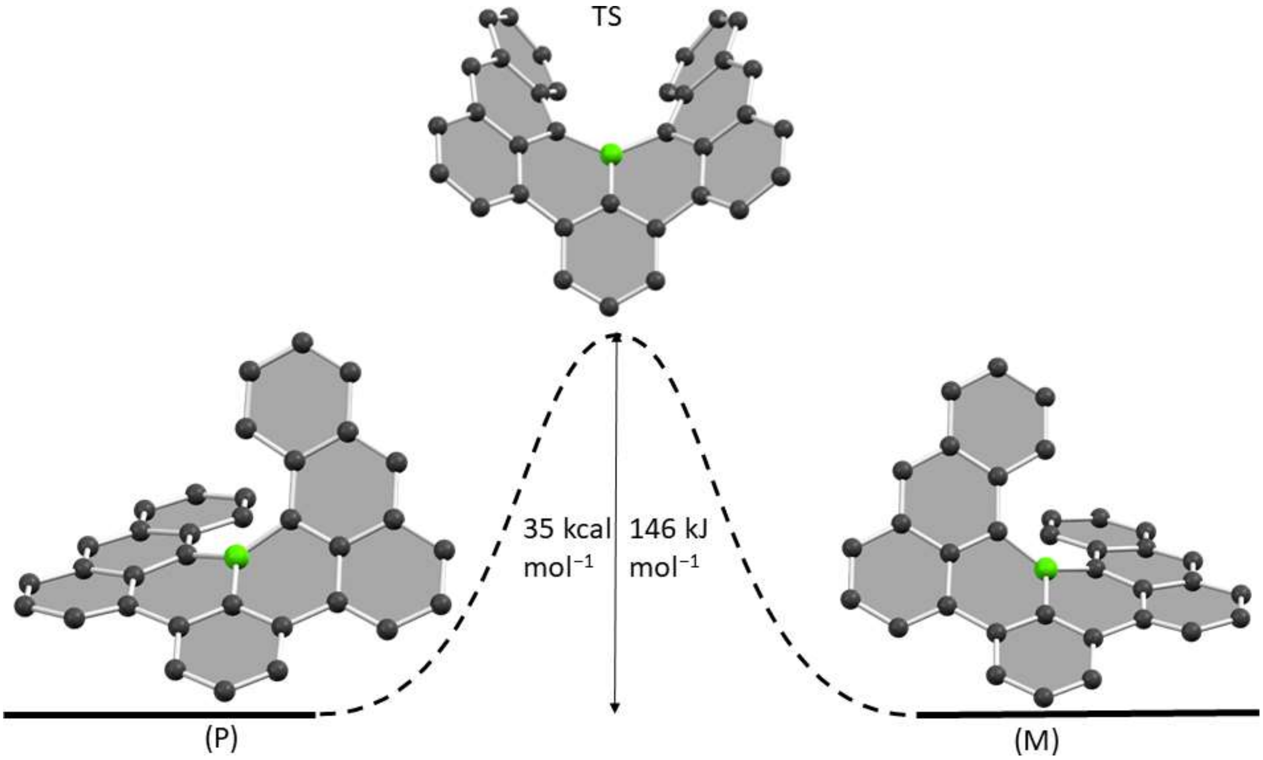

Figure S24. Computational racemization barrier. All calculations at B3LYP/6-311+G(d) level of theory. Transition state obtained with QST3. Barrier is uncorrected.

11.5. Optimized structures

*Cartesian coordinates of the optimized geometry of (P)-**1** at the B3LYP/6-311+G(d,p) level of theory*

C	1.19949800	2.73776300	0.26231800
C	1.18255800	4.13891600	0.25186400
C	-0.00006400	4.82425100	0.00015400
C	-1.18266100	4.13890100	-0.25165600
C	-1.19952700	2.73775500	-0.26229600
C	-0.00000500	2.03583500	-0.00002300
B	0.00000000	0.49988000	-0.00003100
C	-2.43832000	1.97308700	-0.51506400
C	-3.55696900	2.58236500	-1.05039700
C	-4.79417100	1.91261800	-1.19455100
C	-4.92972400	0.62885700	-0.74974800
C	-3.81416400	-0.05851600	-0.18809600
C	-2.52032600	0.57920600	-0.13502300
C	-3.96117000	-1.34977000	0.31877900
C	-2.89757400	-2.03426700	0.91150800
C	-1.58740300	-1.43530200	0.89698700
C	-1.38222300	-0.16210200	0.28474700
C	2.43832200	1.97311500	0.51499200
C	2.52033100	0.57922400	0.13497700
C	3.81417700	-0.05848600	0.18802700
C	4.92976400	0.62891500	0.74958500
C	4.79422800	1.91270100	1.19432100
C	3.55701000	2.58242300	1.05021500
C	1.38222800	-0.16210700	-0.28475700
C	1.58740600	-1.43533000	-0.89695700
C	2.89757600	-2.03429500	-0.91146300
C	3.96117700	-1.34976400	-0.31878800
C	0.54217500	-2.14805900	-1.55606000
C	0.76471300	-3.36122000	-2.15243100
C	2.05157000	-3.95975700	-2.12837500
C	3.08987300	-3.30939800	-1.52366100
C	-0.54216700	-2.14801300	1.55610000
C	-0.76470300	-3.36115200	2.15251500
C	-2.05156400	-3.95968200	2.12849300
C	-3.08986900	-3.30934400	1.52375800
H	2.08870800	4.70740100	0.41681200
H	-0.00008600	5.90886200	0.00024000
H	-2.08885100	4.70735200	-0.41650800
H	-3.50057700	3.61446600	-1.37206300
H	-5.63474000	2.43708600	-1.63425600
H	-5.88137100	0.11203300	-0.81346100
H	-4.93807100	-1.82273800	0.27893700
H	5.88141500	0.11209700	0.81327700
H	5.63481800	2.43720700	1.63393900
H	3.50064600	3.61453800	1.37183700
H	4.93808400	-1.82272100	-0.27894600
H	-0.44261900	-1.70471700	-1.60186900
H	-0.05040800	-3.86967900	-2.65511500
H	2.20644500	-4.92326500	-2.60061700
H	4.08449600	-3.74307300	-1.51290400
H	0.44263000	-1.70467200	1.60187600
H	0.05042000	-3.86960000	2.65520700
H	-2.20644200	-4.92317200	2.60077300
H	-4.08449200	-3.74302100	1.51302200

12. References

[1] G. Seybold, G. Wagenblast, *Dyes and Pigments* **1989**, *11*, 303.
[2] G. R. Fulmer, A. J. M. Miller, N. H. Sherden, H. E. Gottlieb, A. Nudelman, B. M. Stoltz, J. E. Bercaw, K. I. Goldberg, *Organometallics* **2010**, *29*, 2176.
[3] W. Kabsch, *Acta Crystallogr. Sect. D* **2010**, *66*, 125.
[4] G. Sheldrick, *Acta Crystallogr. Sect. A* **2015**, *71*, 3.
[5] G. Sheldrick, *Acta Crystallogr. Sect. C* **2015**, *71*, 3.
[6] A. Spek, *Acta Crystallogr. Sect. E* **2020**, *76*, 1.
[7] M. Frisch, G. Trucks, H. Schlegel, G. Scuseria, M. Robb, J. Cheeseman, G. Scalmani, V. Barone, B. Mennucci, G. Petersson, H. Nakatsuji, M. Caricato, X. Li, H. Hratchian, A. Izmaylov, J. Bloino, G. Zheng, J. Sonnenberg, M. Hada, M. Ehara, K. Toyota, R. Fukuda, J. Hasegawa, M. Ishida, T. Nakajima, Y. Honda, O. Kitao, H. Nakai, T. Vreven, J. Montgomery, J. Peralta, F. Ogliaro, M. Bearpark, E. B. J. Heyd, V. S. K. Kudin, J. N. R. Kobayashi, K. Raghavachari, A. Rendell, J. Burant, S. Iyengar, J. Tomasi, M. Cossi, N. Rega, J. Millam, M. Klene, J. Knox, J. Cross, V. Bakken, C. Adamo, J. Jaramillo, R. Gomperts, R. Stratmann, O. Yazyev, A. Austin, R. Cammi, C. Pomelli, J. Ochterski, R. Martin, K. Morokuma, V. Zakrzewski, P. S. G. Voth, J. Dannenberg, S. Dapprich, A. Daniels, O. Farkas, J. Foresman, J. Ortiz, J. Cioslowski, D. Fox, Gaussian 09 Rev A.2, **2009**.
[8] T. Lu, F. Chen, *J. Comput. Chem.* **2012**, *33*, 580.
[9] P. R. Horn, Y. Mao, M. Head-Gordon, *Phys. Chem. Chem. Phys.* **2016**, *18*, 23067.
[10] Y. Shao, Z. Gan, E. Epifanovsky, A. T. B. Gilbert, M. Wormit, J. Kussmann, A. W. Lange, A. Behn, J. Deng, X. Feng, D. Ghosh, M. Goldey, P. R. Horn, L. D. Jacobson, I. Kaliman, R. Z. Khaliullin, T. Kuś, A. Landau, J. Liu, E. I. Proynov, Y. M. Rhee, R. M. Richard, M. A. Rohrdanz, R. P. Steele, E. J. Sundstrom, H. L. Woodcock, P. M. Zimmerman, D. Zuev, B. Albrecht, E. Alguire, B. Austin, G. J. O. Beran, Y. A. Bernard, E. Berquist, K. Brandhorst, K. B. Bravaya, S. T. Brown, D. Casanova, C.-M. Chang, Y. Chen, S. H. Chien, K. D. Closser, D. L. Crittenden, M. Diedenhofen, R. A. DiStasio, H. Do, A. D. Dutoi, R. G. Edgar, S. Fatehi, L. Fusti-Molnar, A. Ghysels, A. Golubeva-Zadorozhnaya, J. Gomes, M. W. D. Hanson-Heine, P. H. P. Harbach, A. W. Hauser, E. G. Hohenstein, Z. C. Holden, T.-C. Jagau, H. Ji, B. Kaduk, K. Khistyaev, J. Kim, J. Kim, R. A. King, P. Klunzinger, D. Kosenkov, T. Kowalczyk, C. M. Krauter, K. U. Lao, A. D. Laurent, K. V. Lawler, S. V. Levchenko, C. Y. Lin, F. Liu, E. Livshits, R. C. Lochan, A. Luenser, P. Manohar, S. F. Manzer, S.-P. Mao, N. Mardirossian, A. V. Marenich, S. A. Maurer, N. J. Mayhall, E. Neuscamman, C. M. Oana, R. Olivares-Amaya, D. P. O'Neill, J. A. Parkhill, T. M. Perrine, R. Peverati, A. Prociuk, D. R. Rehn, E. Rosta, N. J. Russ, S. M. Sharada, S. Sharma, D. W. Small, A. Sodt, et al., *Mol. Phys.* **2015**, *113*, 184.
[11] J. Contreras-García, E. R. Johnson, S. Keinan, R. Chaudret, J.-P. Piquemal, D. N. Beratan, W. Yang, *J. Chem. Theory Comput.* **2011**, *7*, 625.
[12] W. Humphrey, A. Dalke, K. Schulten, *J. Mol. Graphics* **1996**, *14*, 33.
[13] N. P. Tsvetkov, E. Gonzalez-Rodriguez, A. Hughes, G. dos Passos Gomes, F. D. White, F. Kuriakose, I. V. Alabugin, *Angew. Chem. Int. Ed.* **2018**, *57*, 3651.
[14] Z. Chen, C. S. Wannere, C. Corminboeuf, R. Puchta, P. v. R. Schleyer, *Chem. Rev.* **2005**, *105*, 3842.
[15] M. Antić, B. Furtula, S. Radenković, *J. Phys. Chem. A* **2017**, *121*, 3616.
[16] D. Geuenich, K. Hess, F. Köhler, R. Herges, *Chem. Rev.* **2005**, *105*, 3758.
